# Predicting leukemic transformation in myelodysplastic syndrome using a transcriptomic signature

**DOI:** 10.3389/fgene.2023.1235315

**Published:** 2023-10-25

**Authors:** Chao Guo, Ya-Yue Gao, Zhen-Ling Li

**Affiliations:** Department of Hematology, China-Japan Friendship Hospital, Beijing, China

**Keywords:** myelodysplastic syndrome, AML transformation, expression, WGCNA, LASSO

## Abstract

**Background:** For prediction on leukemic transformation of MDS patients, emerging model based on transcriptomic datasets, exhibited superior predictive power to traditional prognostic systems. While these models were lack of external validation by independent cohorts, and the cell origin (CD34^+^ sorted cells) limited their feasibility in clinical practice.

**Methods:** Transformation associated co-expressed gene cluster was derived based on GSE58831 (‘WGCNA’ package, R software). Accordingly, the least absolute shrinkage and selection operator algorithm was implemented to establish a scoring system (i.e., MDS15 score), using training set (GSE58831 originated from CD34^+^ cells) and testing set (GSE15061 originated from unsorted cells).

**Results:** A total of 68 gene co-expression modules were derived, and the ‘brown’ module was recognized to be transformation-specific (R^2^ = 0.23, *p* = 0.005, enriched in transcription regulating pathways). After 50,000-times LASSO iteration, MDS15 score was established, including the 15-gene expression signature. The predictive power (AUC and Harrison’s C index) of MDS15 model was superior to that of IPSS/WPSS in both training set (AUC/C index 0.749/0.777) and testing set (AUC/C index 0.933/0.86).

**Conclusion:** By gene co-expression analysis, the crucial gene module was discovered, and a novel prognostic system (MDS15) was established, which was validated not only by another independent cohort, but by a different cell origin.

## Introduction

MDS represents a heterogenous cluster of myeloid neoplasms, featured by dysplasia, ineffective hematopoiesis, with or without excessive blasts. Leukemic transformation is one of the main causes of death in MDS patients (Platzbecker et al., 2021). However, evaluation of transformation risk for individual patients, remains an essential but difficult aspect in clinical investigations. By far several prognostic systems have been established, such as IPSS (International Prognostic Scoring System (Greenberg et al., 1997)), IPSS-R (Revised International Scoring System (Greenberg et al., 2012)) and WPSS (WHO Prognostic Scoring System (Malcovati et al., 2007)), which are based on clinical (cytopenia, blast percentage, disease subtypes, etc.) or genetic variables (cytogenetics). The risk categories correlated with duration of leukemic transformation without treatment, according to which the risk-adapted treatment strategy was used in clinical practice. Nevertheless, it is recognized that patients with the same genetic signature (mutation/cytogenetic variation) frequently have distinct clinical and prognostic features. Genes involving in RNA splicing, epigenetic modification and signaling transduction, were most frequently mutated in MDS (Lindsley and Ebert, 2013), which lead to dysregulation of gene expression. Beyond DNA variation, the emerging transcriptomic investigations unraveled the gene expression signature for MDS (Pellagatti et al., 2006; Pellagatti et al., 2010; Gerstung et al., 2015), and a few prognostic models were built. The work of Moritz Gerstung et al. even proved that the predictive power of expression signature was superior to that of traditional markers (IPSS, genetics, cytogenetic, etc.) on AML-free survival (Gerstung et al., 2015). While these studies were lack of external validation by independent cohorts, and the robustness of models was questioned due to the heterogeneity between experimental platforms and cell origins. Additionally, these models were either too elaborate (included too many variables), or derived from CD34^+^ sorted cells, which limited the feasibility in practice (unsorted samples instead of CD34^+^ sorted were used in most clinics).

New methods had emerged to analyze the transcriptomic datasets, which made it possible to improve the accuracy of the prediction model. An updated transcriptomic analysis method, WGCNA, can recognize co-expressed gene clusters according to scale-free network theory and correlated clinical or genetic traits with MEs (the first principal component representing gene co-expression modules) (Zhang and Horvath, 2005; Langfelder and Horvath, 2008). By WGCNA, the co-expressed gene clusters, significantly associating with AML transformation or high-risk factors, were unraveled using GSE58831 dataset in the present study. MDS15 model was established using multiple-iteration LASSO, consisting of 15 gene expression variables. The MDS15 model comprises expression markers from 15 genes: NEAT1, LYSMD2, SLC4A1AP, KMT2A, PHC1, ADHFE1, TFAP2E, TPBG, TRIP11, GAS6-AS1, KCNMB4, ZNF225, LOC100506730, WT1, and STARD9. The NEAT1 gene yields a lncRNA known to influence AML progression (Zhao et al., 2019; Feng et al., 2020; Yan et al., 2021; Rostami et al., 2022). KMT2A also stands as a prognostic determinant in MDS/AML (Li et al., 2013; Kotani et al., 2019), with its partial tandem duplication or rearrangement traditionally recognized as harbingers of diminished survival. GAS6-AS1, another long noncoding RNA, is notably overexpressed in AML and correlates with adverse survival outcomes; curbing its expression has been shown to decelerate AML progression (Zhou et al., 2021). Several studies have illuminated that elevated WT1 expression detrimentally affects the survival rates in MDS, and its expression in peripheral blood astutely forecasts progression-free survival (Rautenberg et al., 2019). However, the remaining genes delineated in the MDS15 model have not been definitively linked to either MDS prognosis or the biology of myeloid malignancies.

**FIGURE 1:**
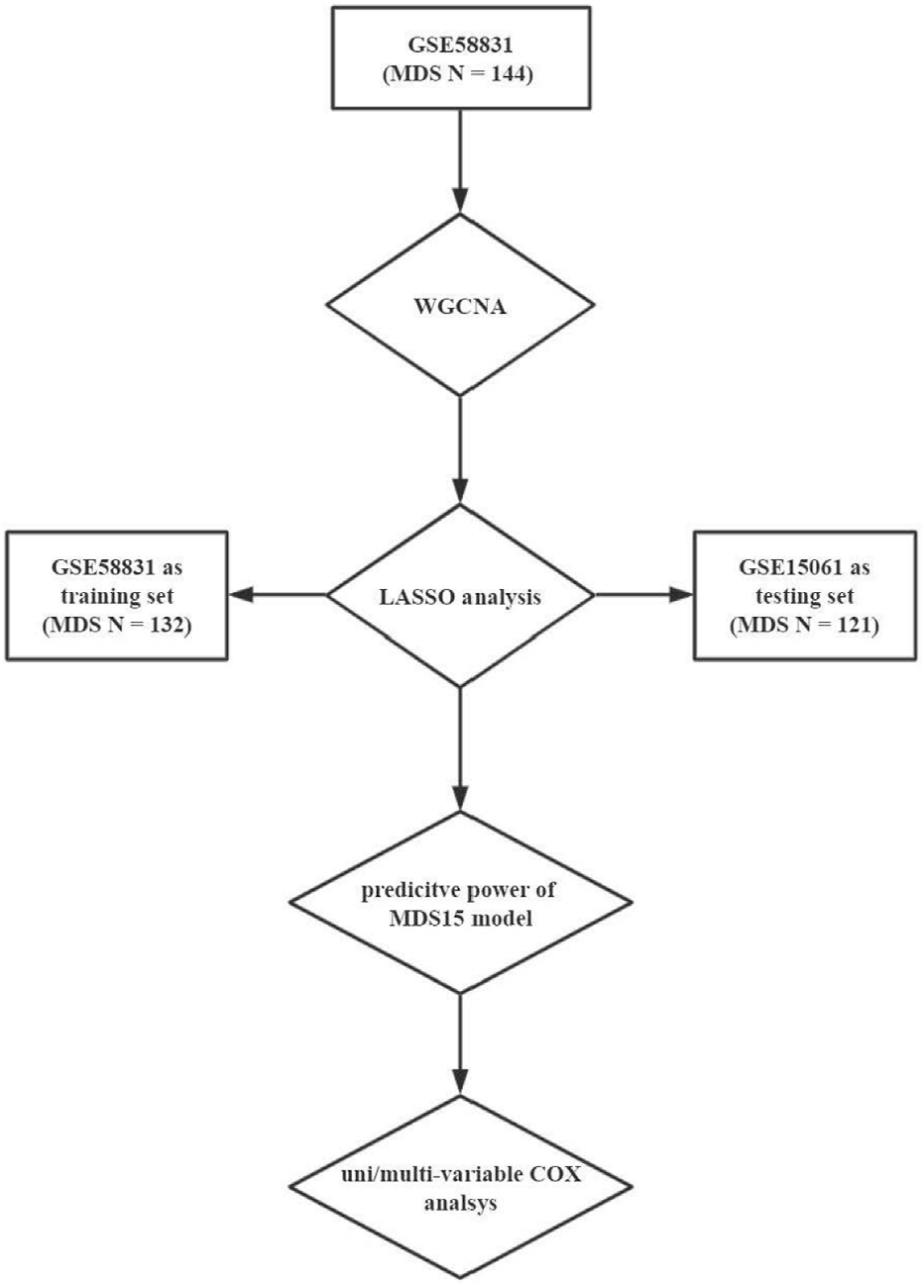
The flowchart of the present study.

Then to address the versatility of MDS15, predictive power was validated in both GSE58831 and GSE15061 datasets. Furthermore, GSEA was implemented to uncover the possible related biological process to MDS15 risk scores. The flowchart of this study was shown in Figure 1.

## Methods

### Datasets download

We searched the GEO database by the following key word: “(myelodysplastic syndrome) AND "*Homo sapiens*" [porgn:__txid9606]”, then “Expression profiling by array” and “Expression profiling by high-throughput sequencing” were selected to limit study type. A total of 164 datasets were obtained at first search, then we selected datasets with corresponding individual leukemic transformation-free survival (LFS) data. Finally, GSE58831 and GSE15061 were selected to be used in our analysis.

The expression matrix and parallel clinical/genetic information were derived from GEO database repository (https://www.ncbi.nlm.nih.gov/gds/) (Edgar et al., 2002). There were 176 bone marrow CD34^+^ cell samples (159 MDS patients and 17 healthy controls) in GSE58831 cohort, with attached individual diagnostic subtypes, karyotypes, hemoglobulin level, count of neutrophils and platelets, IPSS/WPSS scores and LFS. Due to the adequate available individual clinical and genetic data, GSE58831 was selected for WGCNA to draw the AML-transformation specific gene module, which were then inputted into LASSO analysis and establish predictive model as the training set.

GSE15061 cohort included transcriptomic data of 435 bone marrow mononuclear cell samples in total (164 MDS patients and 69 healthy controls, on GPL570 platform), with substantial individual diagnosis, IPSS, blast scores, cytopenia, and LFS information. GSE15061 dataset was used to validate predictive power of MDS15 model, by which the consistency of model performance in CD34^+^ cells and unsorted bone marrow cells will be documented.

Additionally, GSE19429 cohort constituted of 183 MDS and 17 healthy controls with expression matrix and diagnosis information, which was used in correlation analysis between MDS15 scores and MDS subtypes.

All 3 expression datasets (GSE58831/GSE15061/GSE19429), used in our analysis, which were obtained from the same microarray platform (GPL570) to reduce trans-platform heterogeneity. All datasets used in our findings were available from public data repository, which was last visited on 22 Jun 2022.

### WGCNA

The co-expression network was established using microarray data of GSE58831, with ‘WGCNA’ package (Langfelder and Horvath, 2008) and R software (version 4.2.1). The transcriptomic outliers in samples were detected by average linkage of hierarchical clustering (Supplementary Figure S1, 2). Then 0.85 was defined as minimal beta in setting soft threshold (Supplementary Figure S3). The inter-gene Pearson’s coefficients were calculated for inputted matrix, thus establishing TOM (topological overlap matrix). The minimal size of gene modules was set to be 30. Then whole genome was divided into co-expression gene modules by average linkage hierarchical clustering, in which modules with TOM-based dissimilarity less than 0.30 were merged. Module eigengene were defined as the principal component of individual co-expression modules. MM (Module membership, referring to Pearson’s correlation coefficients between individual gene and eigengene in the same module), and GS (gene significance, referring to Pearson’s coefficients between gene expression and clinical/genetic variables) were calculated to elucidate clinical significance of gene clusters. Correlation between target markers (leukemic transformation, etc.) and modules eigengenes, was analyzed to discern the leukemic transformation specific gene cluster (with greatest correlation coefficient).

### Gene enrichment analysis of genes in the selected module

Gene enrichment analysis was performed to demonstrate the possible involved biological process (BP), molecular function (MF), and cell component for leukemic transformation specific gene module, based on DAVID (Huang et al., 2009) (Database for Annotation, Visualization and Integrated Discovery) (https://david.ncifcrf.gov/). The pathways with local FDR adjusted *p*-value (q value) less 0.05, were referred to be significantly enriched by the gene module.

### Prognostic model for leukemia free survival

The detail mathematic transformation of LASSO was described in the original work by Monica M. Vasquez et al. (Vasquez et al., 2016). Normalized expression matrix of brown module within GSE58831 MDS patients, was inputted into LASSO analysis with glmnet package. Then we performed the regression analysis in dimension reduction for inputted variables, and obtained the prediction model consisting of variables with non-zero coefficients after 50,000 times of iteration, which was named as MDS15 due to included gene count. A bootstrap aggregation approach was performed with 10-fold cross validation to fit a binomial regression model, using ‘glmnet’ package. GSE58831 was used as a training set and GSE15061 as a testing set. Then, risk scores of individual patients were calculated to add up weighted expression value of gene variables with non-zero coefficients, which constituted MDS15 model (15 genes included). The cutoff value of low and high-risk groups was determined with function ‘surv_cutpoint’ within package ‘survminer’. Kaplan-Meier analysis on LFS and time-dependent ROC were performed with ‘survival’ and ‘survivalROC’ packages in R software. To investigate the relationship of MDS15 risk scores and traditional disease markers, MDS patients was grouped by diagnostic subgroups, with or without cytopenia, IPSS/WPSS category, based on GSE58831/GSE15061/GSE19429 cohorts, respectively. Then MDS15 scores were compared across different subgroups.

Furthermore, the independent prognostic value of MDS15 scores was validated by univariate and multivariate Cox analysis along with other possible prognostic factors (age, gender, MDS subtype, counts of blood cells and blasts, serum ferritin, karyotype scores, IPSS and WPSS).

### Genome-wide expression profile associated with MDS15 scores

To elucidate the associated gene expression signature and pathway profile, genome-wide expression correlation analysis was implemented. Pearson’s coefficients were calculated between MDS15 risk score and expression value of each gene within the genome. R software (version 4.0.2) and ‘stats’ package was utilized for the calculation. Then, GSEA was used to interpret the result of genome-scale correlation analysis for MDS15 score, based on the Pearson’s coefficient of individual gene in the specific sets (pathways) of MSigDB database (Mootha et al., 2003; Subramanian et al., 2005; Dweep et al., 2013). (http://software.broadinstitute.org/gsea/msigdb). The MDS15 related pathways were identified as |NES (normalized enrichment score) | > 1 and q value (FDR adjusted *p*-value) < 0.05.

### Statistical analysis

After download from database, ‘normalizeBetweenArrays’ function of ‘limma’ package was implemented to normalize gene expression data for the following analysis (R software, version 4.2.1). Continuous variables of subgroups were compared by two-side Wilcoxon’s test (N = 2), or Kruskal–Wallis’s test (N > 2). LFS difference between subgroups was discerned by Kaplan-Meier plotter and log-rank test.

## Results

### WGCNA

The clinical and genetic characteristics of GSE58831 cohort were previously described (Mills et al., 2009). Among the GSE58831 cohort, 7 patients with AML transformation at baseline (blast % > 20%) and 7 patients with CMML were excluded in WGCNA. 1 patient (GSE1420528) was excluded by outlier detection (Supplementary Figure S1, 2). A total of 144 MDS patients were included in the WGCNA, consisting of 13 MDS-RA, 6 5q-syndrome, 49 MDS-RCMD, 20 MDS-RARS and 56 MDS-RAEB patients.

**FIGURE 2:**
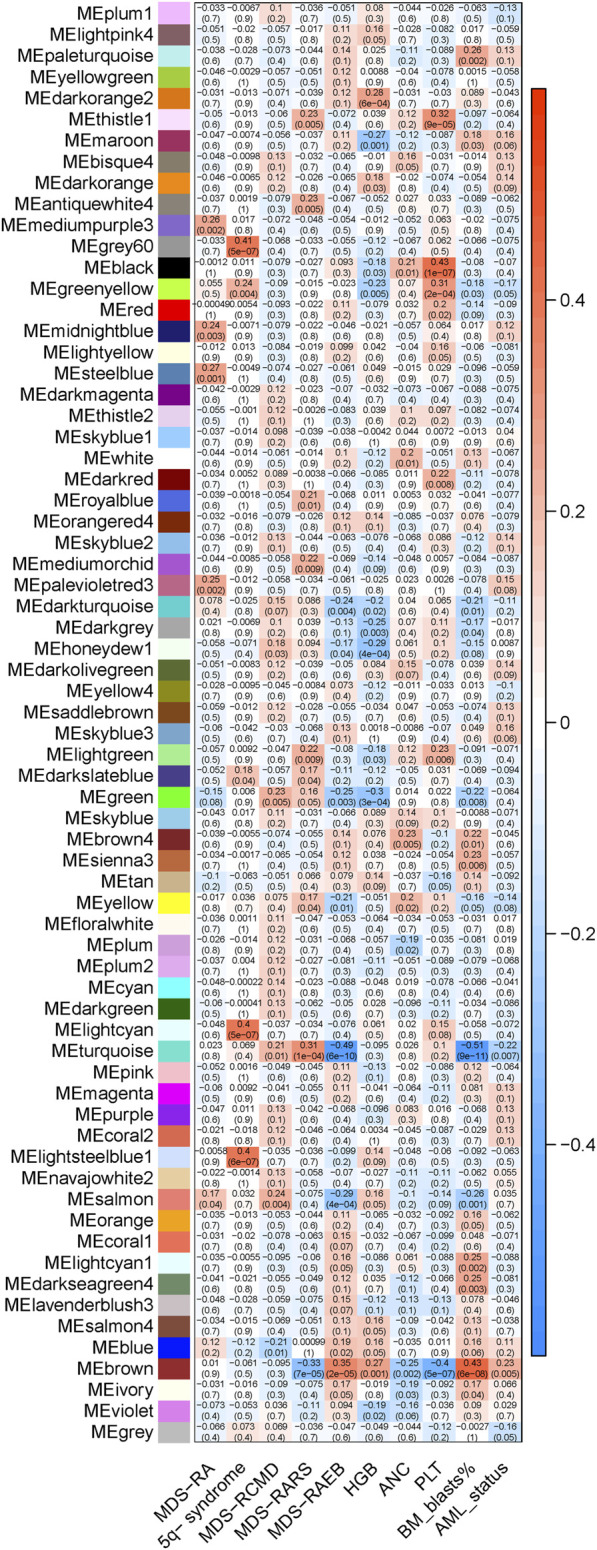
The relationship of module eigengenes and clinical variables. The heatmap displays various modules (represented by distinct colors) on the X-axis, juxtaposed against the evaluated variables on the Y-axis. Each cell within the heatmap provides two key metrics: the top number denotes the Pearson’s correlation coefficient between module eigengenes and evaluated variables, while the bottom indicates the corresponding *p*-value. The color gradient serves to visually convey the correlation’s strength and direction. A shift towards red implies a stronger positive correlation, while a move towards blue denotes a stronger negative correlation.

The minimal soft threshold power was 7, by which the scale topology model fit R^2^ > 0.85 (Supplementary Figure S3). The argument ‘mergeCutHeight’ was set to be 0.30, which merged the modules with TOM-based dissimilarity <30%. Finally, 68 gene clusters/modules were derived (Supplementary Figure S4). The relationship of clinical variables and gene module eigengenes were shown in Figure 2, by which the brown module was recognized as AML transformation specific module (R^2^ = 0.23, *p* = 0.005). Notably, brown module was negatively associated with RARS subtype, neutrophil, and platelet count, while positively associated with RAEB subtype, hemoglobulin and blast percentage in bone marrow (*p* < 0.05, Figure 2). The MM and GS of individual genes in brown module were significantly correlated (R^2^ = 0.31, p = 2e-30, Supplementary Figure S5).

### Over-representation analysis for the brown module

**FIGURE 3:**
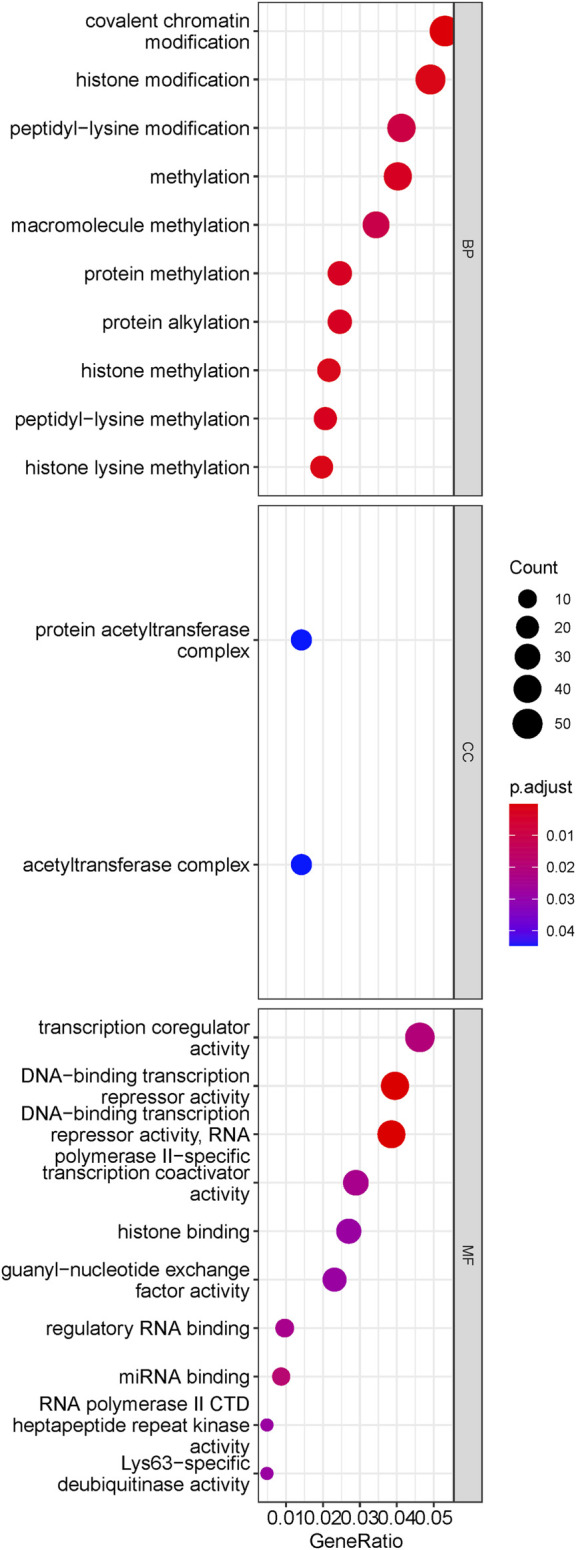
Results of ORA for transformation-specific gene module. The dotplot of enriched pathways. The size of dots represented the count of genes involved in the pathway. while the color of dots correlated with the -log10 (q value). It is indicated that the brown module genes mainly implicated in epigenetic regulation.

The brown module included 1,301 genes, including ASXL1 (Gelsi-Boyer et al., 2009; Mills et al., 2009), ASXL2 (Li et al., 2017), ATR (Nguyen et al., 2018), CUX1 (An et al., 2018; Aly et al., 2019), DNMT3A (Walter et al., 2011), FLT3 (Daver et al., 2019), HOXA7 (Drabkin et al., 2002) and WT1 (Rautenberg et al., 2019), which are dysregulated and/or prognostic in MDS or AML. The results of ORA for genes in the ‘brown’ module demonstrated enriched pathways in Figure 3. Biological processes were significantly enriched, such as covalent chromatin modification, histone modification, methylation histone methylation, etc. And protein acetyltransferase complex was enriched cell component for brown module genes. Moreover, GO analysis on molecular function enrichment, indicated the ‘brown’ module genes were enriched in transcription coregulator activity, DNA-binding transcription repressor activity, histone binding, etc. The above results suggested the brown module were predominantly involved in transcriptional regulation by epigenetic mechanisms.

### LASSO analysis and MDS15 model

LASSO penalized regression analysis fitted LFS of individual patients to establish prediction model, by inputting expression level of brown module genes. GSE58831 was used as the training cohort, including 121 patients with sufficient LFS data. And GSE15061 was used as the testing cohort, including 132 patients with sufficient LFS data.

After 50,000-times iteration, an optimized model of 15 gene with non-zero coefficient were derived, balanced predictive power in both training and testing cohorts. The prediction model (MDS15) was included 15 genes and matched coefficients (listed in Table 1). The risk scores were calculated by summation of individual weighted gene expression value.

**TABLE 1:** The arguments of MDS15 model, including the gene symbols and corresponding coefficients.

Gene symbol	Coefficient
NEAT1	0.309795
LYSMD2	0.370893
SLC4A1AP	0.404787
KMT2A	0.468553
PHC1	0.219551
ADHFE1	0.093212
TFAP2E	0.532325
TPBG	0.032962
TRIP11	0.00916
GAS6.AS1	0.342155
KCNMB4	−0.71229
ZNF225	−0.08073
LOC100506730	−0.1886
WT1	0.213879
STARD9	−0.23934

### Prediction power of MDS15 model on leukemic transformation

**FIGURE 4:**
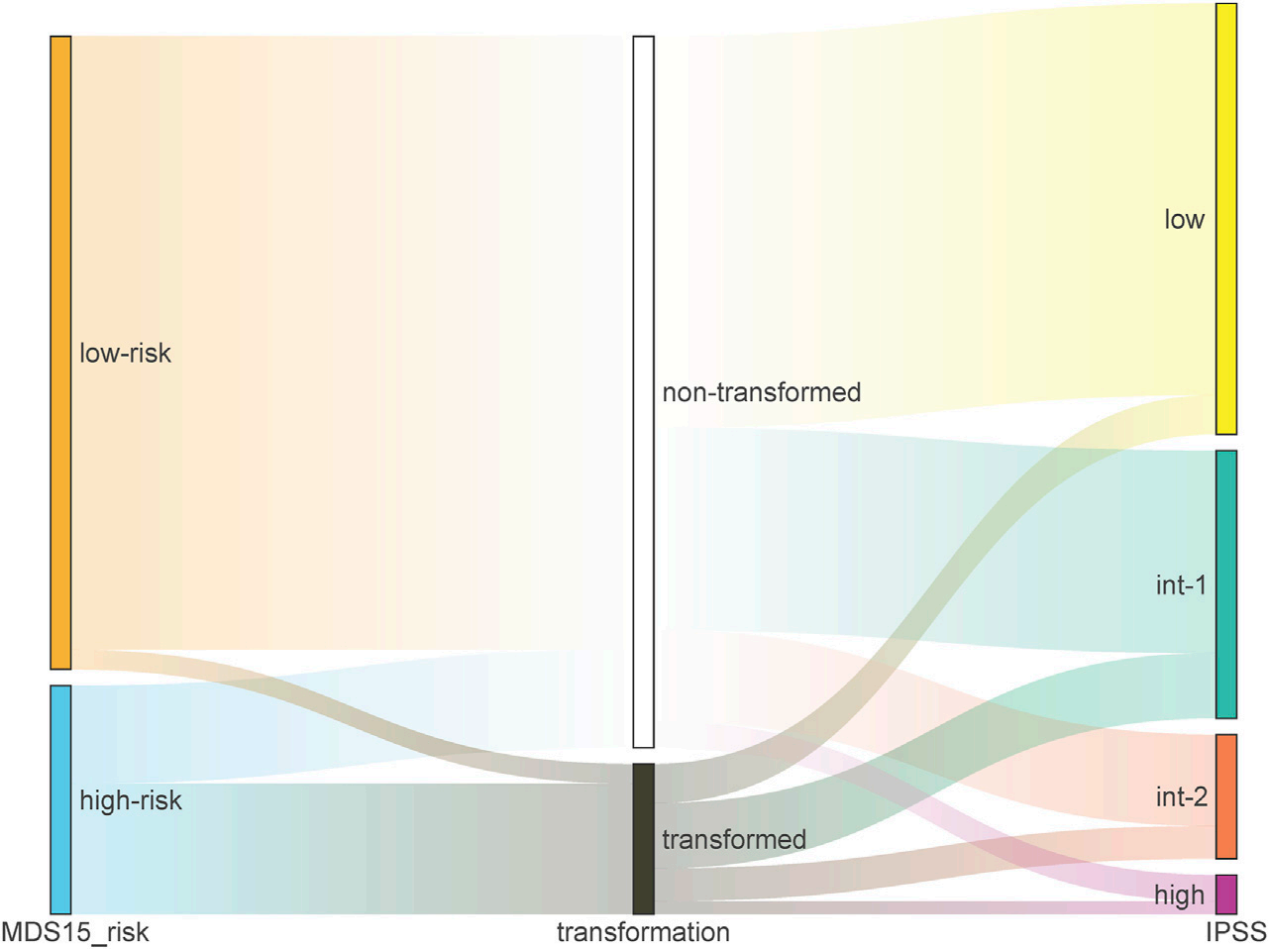
The Sankey plot illustrating the relative precision of prediction by MDS15 model (left column) vs. IPSS (right column) based on GSE15061 dataset, which showed superior predictive power of MDS15 model.

The cohort-specific cut-off risk score was calculated for training and testing cohorts respectively by “surv_cutoff” function of “survminer” package, which stratified the training cohort (low-risk N = 107, high-risk N = 14) and testing cohort (low-risk N = 96, high-risk N = 36). The Sankey plots demonstrated that the capability of current prognostic systems (IPSS/WPSS), was inferior to that of MDS15 model (Figure 4; Supplementary Figure S6A, B). A subset of actually high-risk patients was ignored and classified as very low risk or low risk group in IPSS/WPSS, while the majority of very high-risk group (defined by IPSS/WPSS) had not progressed into AML (Figure 4, Supplementary Figure S6A, B).

**FIGURE 5:**
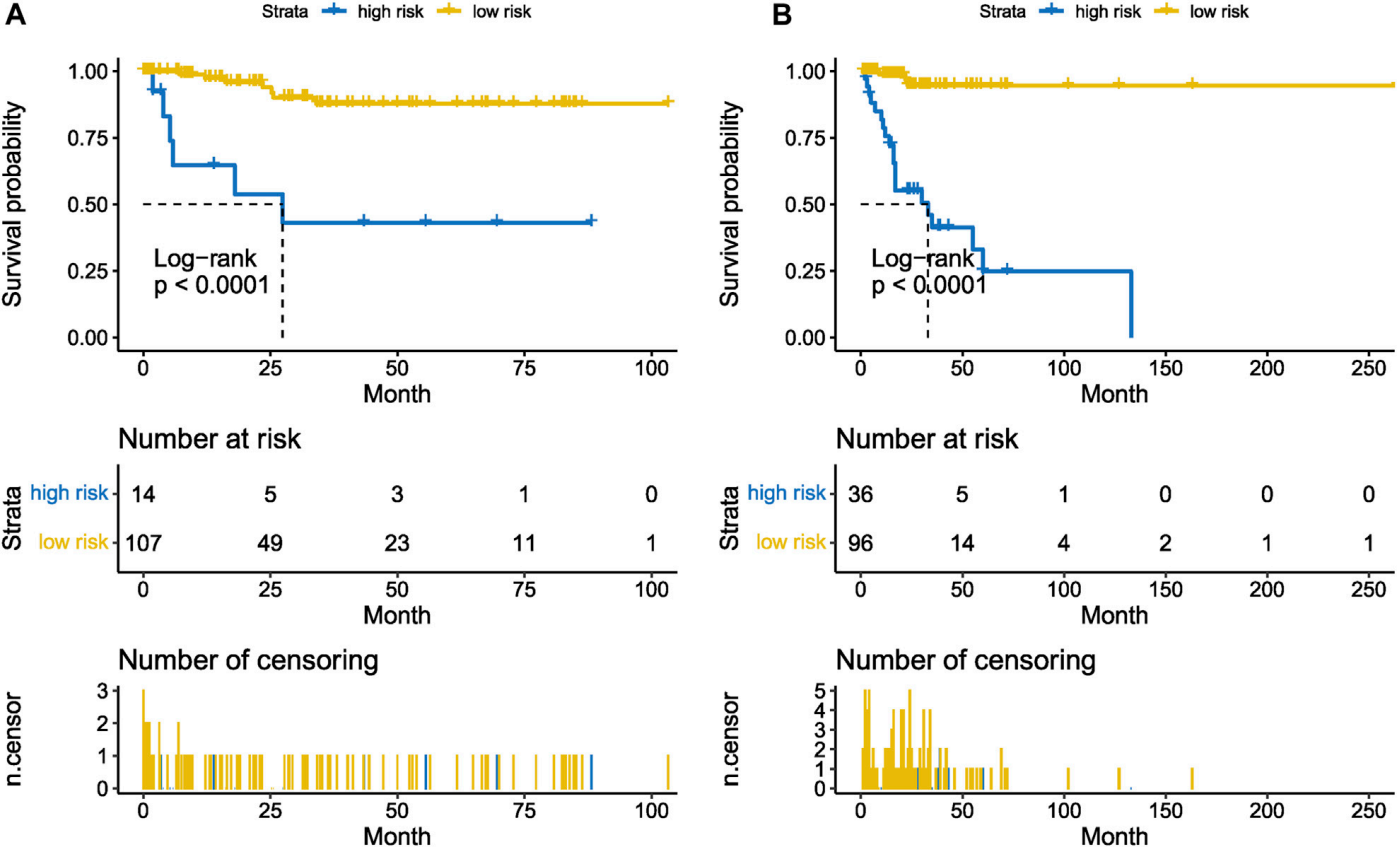
Leukemia-free survival analysis based on MDS15 model by Kaplan-Meier plotter, for the training set (GSE58831, **(A)** and testing set (GSE15061, **(B)**. The MDS15 low-risk group exhibited markedly prolonged survival compared to the MDS15 high-risk group.

**FIGURE 6:**
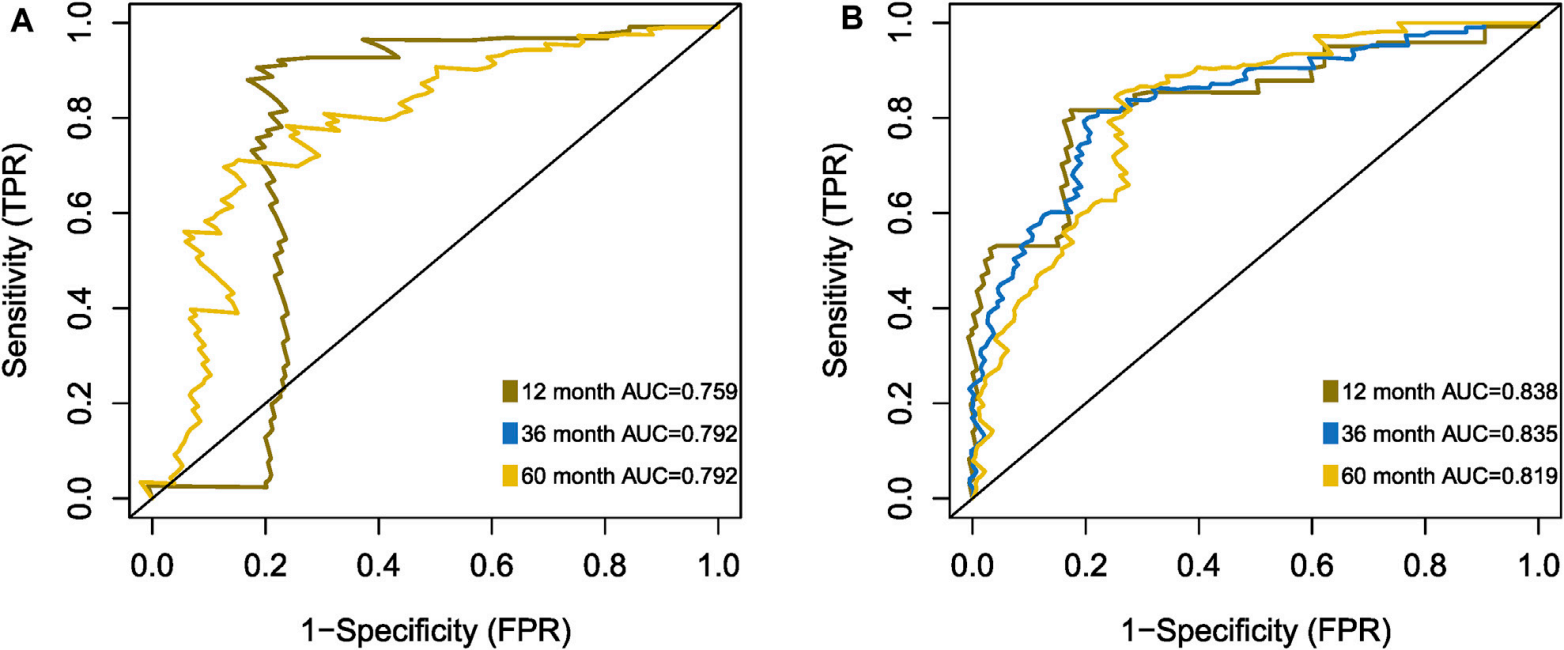
Time-dependent ROC analysis of MDS15 model, based on training set **(A)** and testing set **(B)**.

**FIGURE 7:**
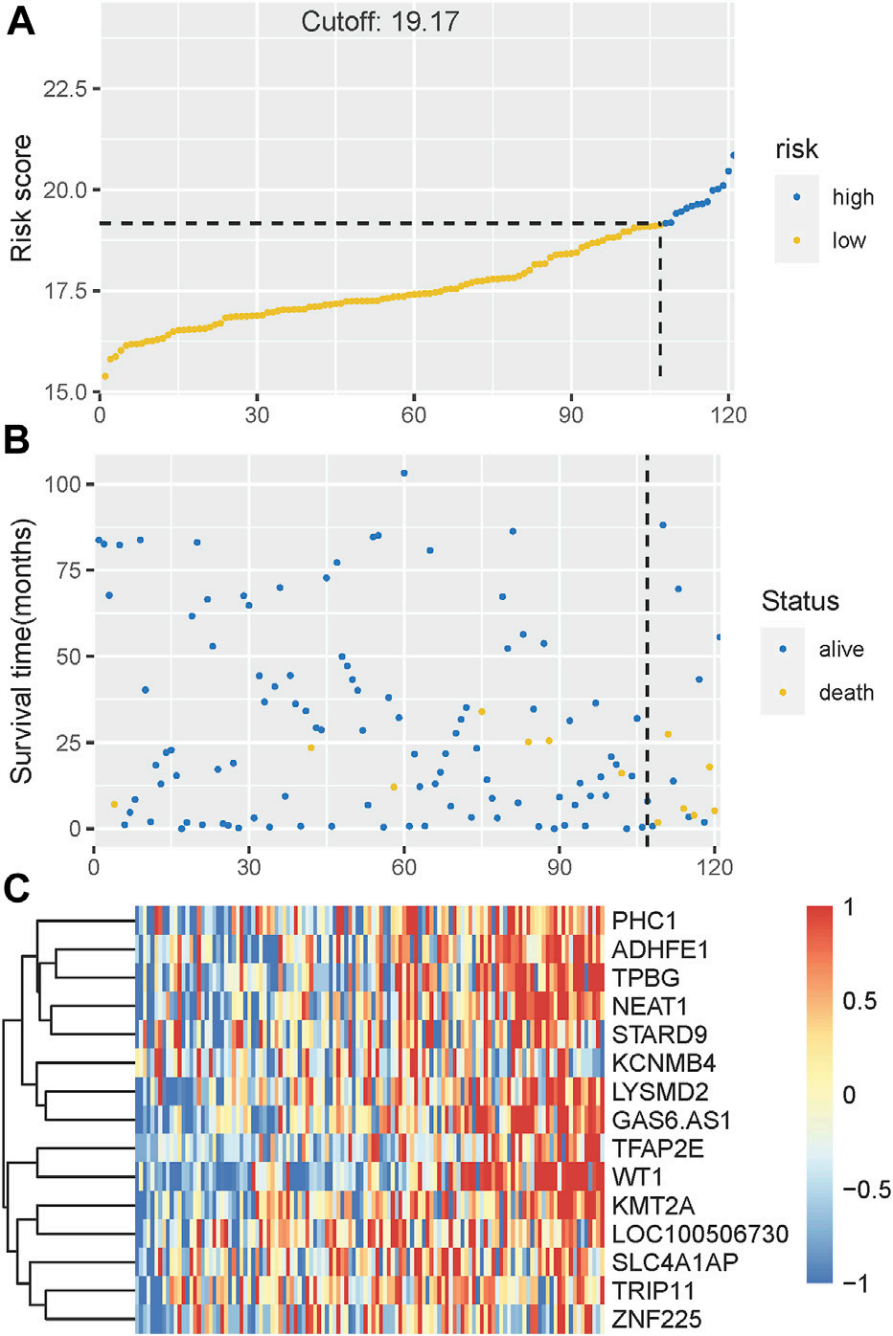
**(A)** The distribution and cut-off value of MDS15 risk scores in the training set. **(B)** The survival time and status of the training set corresponding to risk scores. The left half, separated by a line of dashes, included low-risk group, while the right part included high-risk group. **(C)** The heatmap and hierarchical clustering of the MDS15 model for the training set. The symbols of genes are displayed on the right longitudinal axis; the clustering dendrogram of genes are displayed on the left longitudinal axis. The relative expression level of genes is indicated by gradient color from blue (−1) to red (1).

**FIGURE 8:**
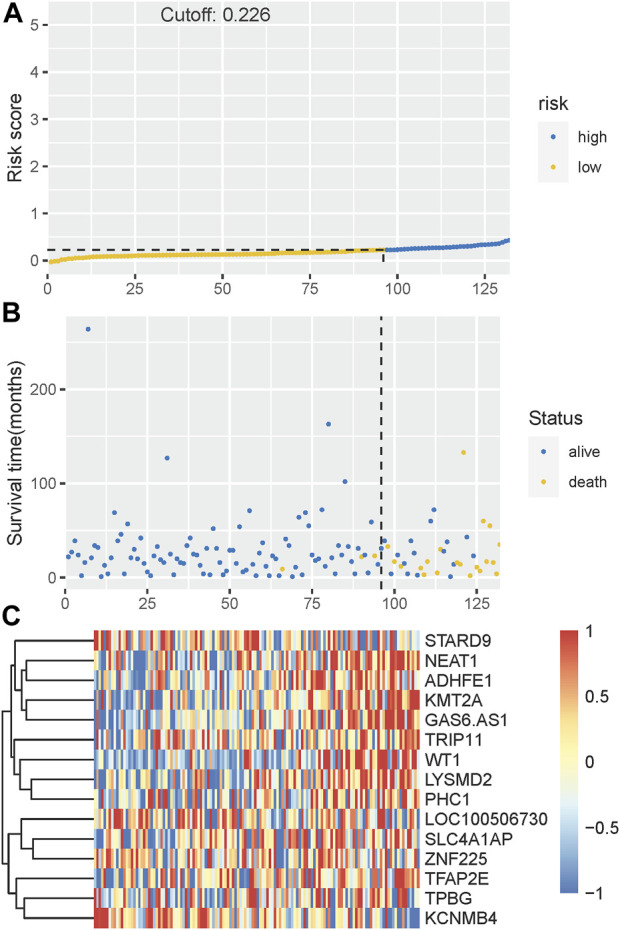
**(A)** The distribution and cut-off value of MDS15 risk scores in the testing set. **(B)** The survival time and status of the training set corresponding to risk scores. The left half, separated by a line of dashes, included low-risk group, while the right part included high-risk group. **(C)** The heatmap and hierarchical clustering of the MDS15 model for the testing set. The symbols of genes are displayed on the right longitudinal axis; the clustering dendrogram of genes are displayed on the left longitudinal axis. The relative expression level of genes is indicated by gradient color from blue (−1) to red (1).

The Kaplan-Meier plotter on LFS of MDS15-high and low risk groups were depicted in Figure 5A&B for training and testing cohorts, respectively, which indicated that the MDS15-high risk groups had significantly shorter LFS than that of MDS15-low risk group (*p* < 0.001 in both cohorts). MDS15 risk stratification well recognized a group of virtually low-risk patients, the median LFS of which was not reached in both cohorts. Moreover, time dependent ROC analysis revealed 12/24/36-month AUC of MDS15 model in training cohort was 0.759/0.792/0.792 (Figure 6A), and that of testing cohort was 0.838/0.835/0.819 (Figure 6B), respectively. The distribution of survival and risk profile was visually exhibited in Figures 7, 8 for the training/testing cohorts, which demonstrated the consistency of MDS15 risk scores and LFS distribution.

**FIGURE 9:**
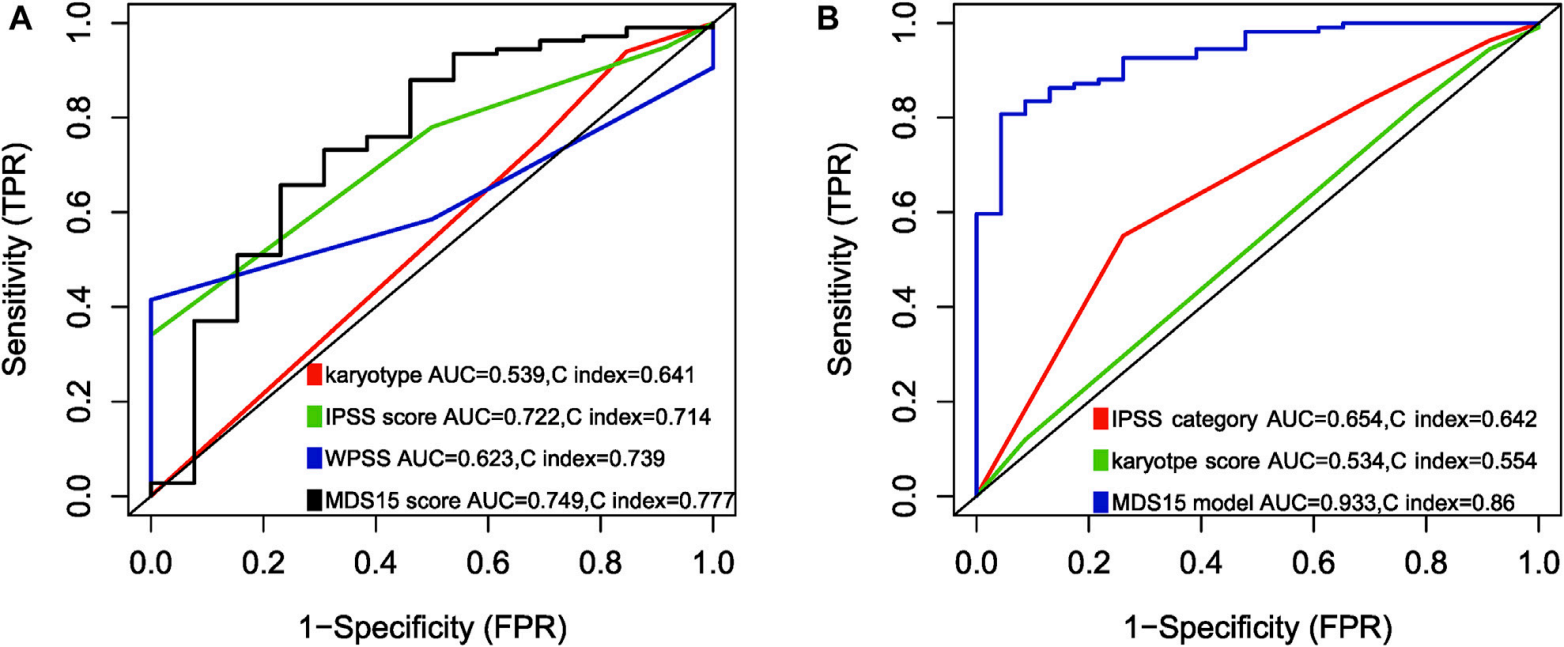
Multi-ROC analysis comparing predictive power (AUC and Harrison’s C index) between MDS15 model and previously used prognostic systems, in training set **(A)** and testing set **(B)**. The parameters of the MDS15 model surpassed those of traditional prognostic systems.

Notably, to compare the prediction power of MDS15 model and traditional MDS risk stratification system, multi-ROC analysis was implemented. The results indicated that AUC and C-index (Harrel’s C statistic) of MDS15 model was superior to karyotype, IPSS and WPSS, validated in the training and testing cohorts (Figures 9A, B).

### The association of MDS15 model with traditional disease markers of MDS transformation

**FIGURE 10:**
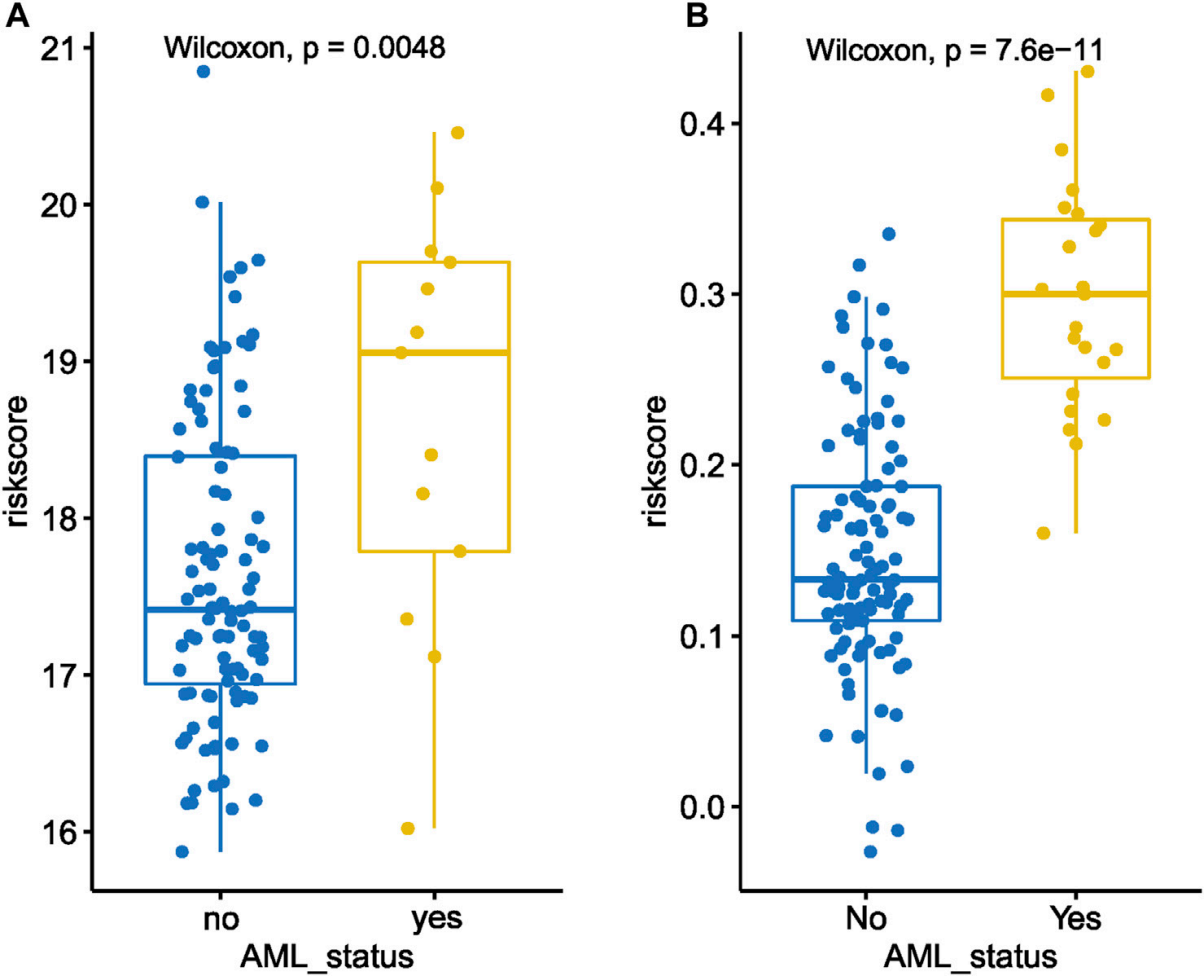
The dot plot illustrates a comparison between the baseline MDS15 risk scores of patients who underwent transformation *versus* those who did not, in GSE58831 **(A)** and GSE15061 **(B)**. Significant difference in risk scores was revealed in both sets.

The correlation analysis indicated transformed MDS patients had higher baseline MDS15 scores than that of non-transformed MDS patients (Figure 10A, B). Moreover, other negative disease markers (RAEB phenotype, cytopenia, higher IPSS/WPSS risk category, higher blast scores, etc.) was also associated with MDS15 risk scores (Supplementary Figure S7A–K), based on 3 independent cohorts (GSE15061/58831/19429).

### Univariate and multivariate cox analysis

**FIGURE 11:**
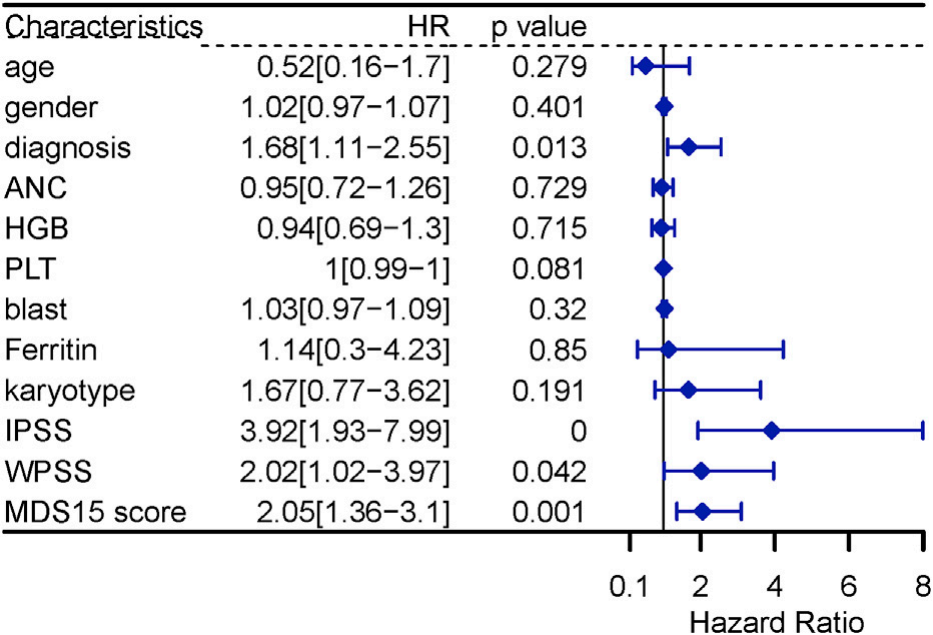
univariable Cox regression analysis for LFS in MDS.

**FIGURE 12:**
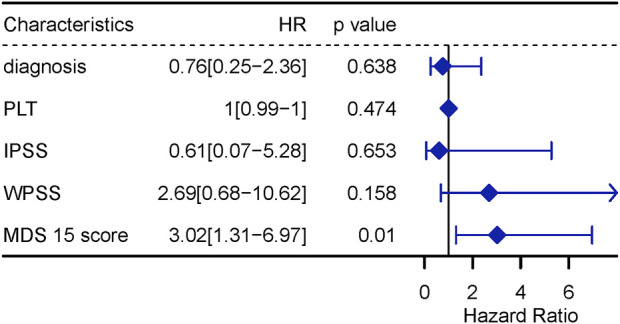
multivariable Cox regression analysis for LFS, including variables with *p* < 0.1 in univariable Cox analysis.

The univariate Cox analysis screened 5 variables associated with LFS (*p* < 0.10), to input into sequential multivariate analysis, including diagnosis, count of platelets, IPSS, WPSS and MDS15 risk scores. Then, multivariable Cox analysis demonstrated MDS15 risk score to be the only independent prognostic factor for LFS of MDS patients (Figure 11, 12).

## Results of genome-wide correlation analysis

**FIGURE 13:**
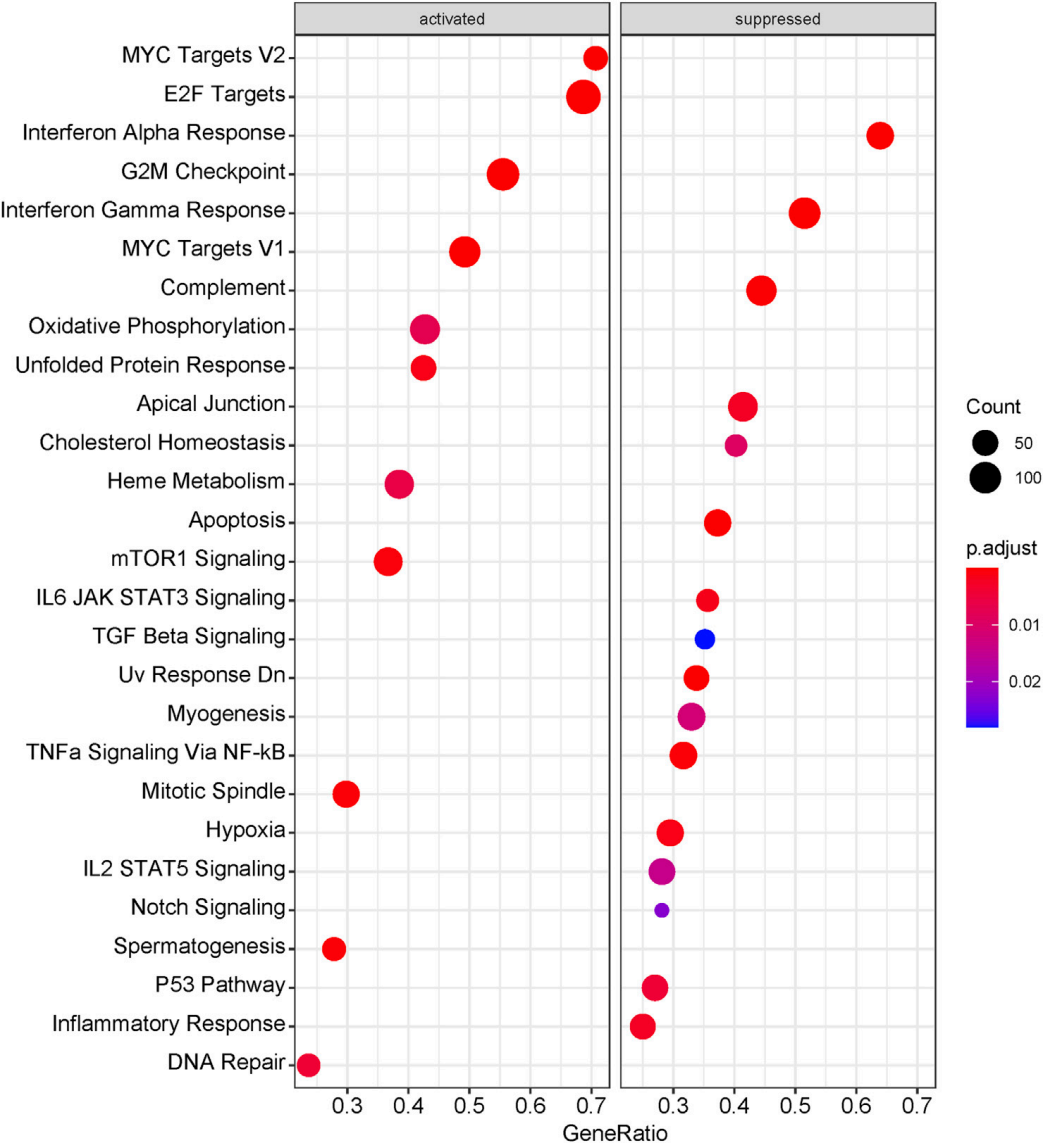
The dotplot of GSEA results associating with MDS15 scores. The size of dots represented the count of genes involved in the corresponding pathways. while the color of dots correlated with the -log10 (adjusted *p*-value).

The significantly enriched pathways relating with MDS15 scores were illustrated in Figure 13. The activated pathways consisted of MYC (Myc proto-oncogene protein) targets, E2F (Transcription factor E2F1) targets, Oxidative Phosphorylation and DNA repair pathways, *etc.*, significantly suppressed pathways constituted apoptosis, IL6-JAK-STAT3 pathway, IL2-STAT5 pathway, Interferon alpha response pathway, apoptosis and p53 pathway, etc.

## Discussion

The transcriptomic features have been widely investigated for bone marrow samples of MDS patients, to link the mRNA expression signature with clinical outcomes or eligibility of target therapy (Pellagatti et al., 2006; Pellagatti et al., 2010; Kondo et al., 2011; Heinrichs et al., 2013; Xu et al., 2016; Huang et al., 2019). The updated analytic methods have provided new clues on the ‘old data’. WGCNA has been used in digging from omics data of hematological malignancies (Fu et al., 2020; Liu et al., 2020; Adebayo et al., 2022; Chen et al., 2022). In comparison with traditional differential expression analysis, WGCNA focused on the interplay of co-expressed modules based on scale-free network theory, so that the gene clusters instead of individual genes were investigated to establish complicated, but robust correlation with clinical variables.

The current MDS risk stratification systems profiling survival and leukemic transformation, were challenge by emerging omics data, including genomics, epigenetics (methylation or chromosomal accessibility), transcriptomic, and proteomics. The omics data profiled the actual MDS cell status and further predict prognosis or discern therapeutic targets. In the present study, the co-expressed gene modules were recognized, among which the brown module was identified as the key gene cluster associating with AML transformation and high-risk clinical factors (RAEB phenotype, etc.. 1,301 genes constituting brown module (Supplementary Table S1), was demonstrated to be predominantly enriched in epigenetic regulating pathways, by biological process (BP) analysis based on GO database. One node of this pathway in brown module, KMT2A gene encodes histone methyltransferase, which plays an essential role in hematopoiesis (Nakamura et al., 2002; Dou et al., 2005; Patel et al., 2009; Avdic et al., 2011), rearrangement or abnormal expression of which had been linked to poor prognosis (Tsai et al., 2022). Other histone methylating genes, such as KMT5B/EZH1/KMT2D/KMT2E/SETD5, were also included in brown module, and related to pathological process or prognosis of myeloid neoplasms (MDS/AML) (Mochizuki-Kashio et al., 2015; Jin et al., 2020; Liquori et al., 2020; Janusz et al., 2021; Lin et al., 2021; Zhong et al., 2021).

MDS15 model was derived from LASSO analysis based on expression signature of brown module, and greatly improved the prediction of leukemic transformation (Figure 3). For GSE15061, only 3.09% (3/97) MDS15 low-risk patients transformed eventually, while 9.09% (6/66) of IPSS low-risk group, 24.39% (10/41) of IPSS int-1 group, progressed into AML eventually. Moreover, 57.14% (20/25) patients in MDS15 high-risk group finally transformed, superior to the result of IPSS (26.32% and 33.33% transformation rate in IPSS int-2 and high-risk group, respectively). Identical results were found in GSE58831, irrespective of MDS15 vs. IPSS (Supplementary Figure S6A) or MDS15 vs. WPSS (Supplementary Figure S6B). The demonstratable advantage in distinguishing actually risky patients in Kaplan-Meier analysis (Figure 5A, B) confirmed prognostic value of MDS15 model.

Time-dependent ROC analysis revealed 12/24/36-month AUC were 0.759/0.792/0.792 for training set, and 0.838/0.835/0.819 for testing set, respectively (Figure 6A, B). The riskscore-survival curves were steady, continuous, and well-fitted in both sets. Notably, GSE58831 and GSE15061 were not only sampled from independent cohorts, but also from different cell origins (GSE58831 derived from CD34^+^ bone marrow cells, GSE15061 from unsorted bone marrow cells). Robust and comparable predictive power was exhibited by both datasets, suggesting MDS15 model bridged between CD34^+^ sorted and unsorted samples. Since only unsorted samples were available in most hospitals or clinics, MDS15 is a promising risk system in clinical practice. In comparison with traditional prognostic scoring systems, superior predictive power (AUC and Harrison’s C index) of this model was discerned by both training and testing set (Figures 9A, B). Transformed patients had significantly higher baseline MDS15 scores (Figure 10A, B). Moreover, the independent prognostic variables were screened in covariate Cox analysis, in which diagnostic subtype, IPSS, WPSS and MDS15 scores were identified to be significant prognostic factors. While only MDS15 scores were demonstrated to be an independent prognostic factor by multi-Cox analysis. The abovementioned results elucidated the prognostic power and future potential in clinical practice.

In spite of the rapid advancement of omics techniques in the realm of myeloid neoplasms, including RNAseq and microarray, few findings have been integrated into clinical practice owing to the prohibitive costs and laboratory requirements. The MDS15 model offers precise prognostic predictions and reduces costs by incorporating fewer variables, rendering it practical for clinical applications.

**FIGURE 14:**
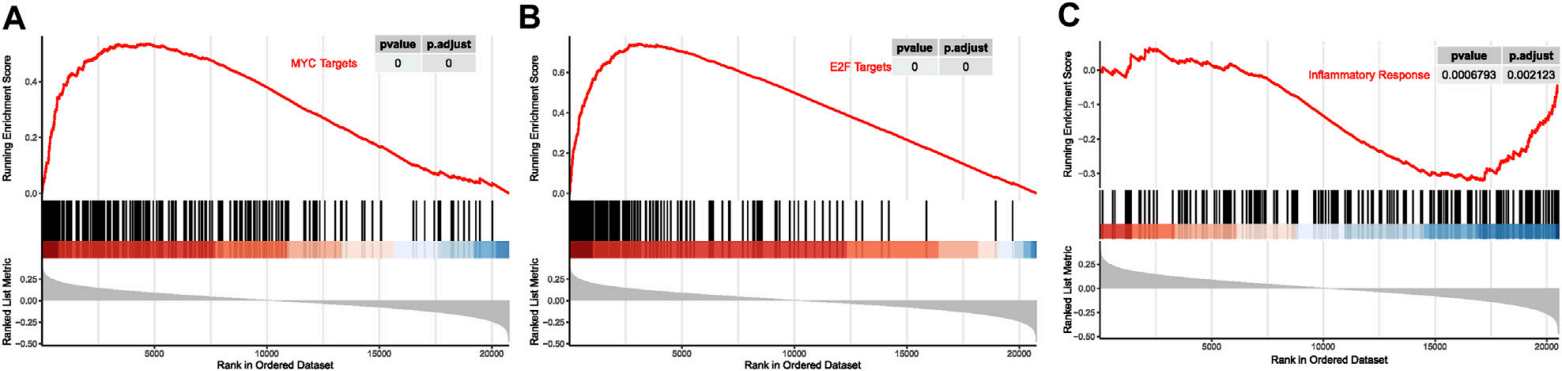
The curves of running enrichment score for MDS15-related pathways, for MYC targets **(A)**, E2F targets **(B)**, and inflammatory response **(C)**.

Moreover, the whole-genome expression analysis profiled cell signaling pathways in relation with MDS15 scores, combining with GSEA. The transcript factors, such as MYC and E2F signaling was activated along with ascending MDS15 scores (core enrichment of which included MYC, TRAF6 and EZH2) (Figure 14). Overexpression of MYC was associated with early AML progression in MDS patients (Gajzer et al., 2021). One of the MYC regulating genes in both core enrichment and brown module, TRAF6 (TNF receptor-associated factor 6 protein) is generally overexpressed in MDS and was attributed to hematopoietic progenitor cell defects, but its expression declined and dysfunction in a subset of AML patients (Muto et al., 2022). Further investigation indicated TRAF6 mediates ubiquitination of MYC protein and plays a role of tumor suppressor in myeloid neoplasms (Muto et al., 2022), suggesting the decreasing gradient of TRAF6 expression was related to leukemic transformation risk. Consistent with this result, expression of TRAF6 was significantly negatively correlated with MDS15 risk scores (*p* = 0.02, Pearson’s coefficient = −0.21, Supplementary Figure S8).

EZH2 is a downstream target of E2F signaling (Bracken et al., 2003; He et al., 2019), the loss-of-function mutation of which is an independent prognostic factor for MDS. Despite of EZH2 expression declines in loss of 7q karyotype, EZH2 or SFSR2 mutant patients (Nagata and Maciejewski, 2019; Sakhdari et al., 2022), the expression profile in general MDS patients had not been investigated. After we dichotomized GSE58831 cohort into EZH2-high and EZH2-low expression subgroups by the median expression level, a trend toward LFS difference was found in Kaplan-Meier plot (log-rank *p* = 0089, Supplementary Figure S9A). Considering that harboring 7q abnormality (−7 or del7q) is an independent prognostic factor and lead to haplo-insufficiency of EZH2 (located in 7q36), 5 such cases were then excluded in LFS analysis, resulting a significant LFS difference between EZH2-high and EZH2-low subgroups (log-rank *p* = 0.0017, Supplementary Figure S6B). The expression of EZH2 was also correlated with MDS15 risk scores (*p* = 0.003). Abovementioned results indicated the transcriptomic signature of oncogenic signaling (e.g., MYC/E2F), associating with MDS15 scores, contributed to tumor cell proliferation and disease progression.

Notably, inflammatory signaling pathways (interferon alpha/gamma signaling, TNF alpha signaling via NF-kB, inflammatory response, etc.) were suppressed when the risk score increased (Figures 13, 14). The interplay between inflammatory cytokines and MDS stem cells was complicated and paradoxical (Hemmati et al., 2017). Both interferon alpha and gamma induce apoptosis and cell growth inhibition in hematopoietic cells (Snoeck et al., 1994; Mayer et al., 2001). TNF alpha was reported to be upregulated in bone marrow plasma and positively correlated with cell apoptosis in low-risk MDS (Kerbauy and Deeg, 2007). While inflammation signaling (e.g., TLR signaling) is specific in low-risk MDS, which promotes tumor cell death (Paracatu and Schuettpelz, 2020). These results suggested that suppression of inflammatory signaling attenuated cell death in MDS, which was in favor of leukemic transformation. MYD88 and TLR2, in the core enrichment gene list of inflammatory response signaling, was previously reported to be associated with low-risk MDS, while downregulated in high-risk MDS (Dimicoli et al., 2013; Wei et al., 2013). MYD88 is a downstream mediator following Toll-like receptor activation, the mRNA of which was reported to be over-expressed in CD34^+^ cells of low-risk MDS compared to healthy donors in previous investigation, inhibition of which restored erythroid colony formatting capacity of HSPC. Nevertheless, higher expression level of MYD88 was displayed in high-risk MDS than that in low-risk MDS (Dimicoli et al., 2013). Consistently, expression of MYD88 was significantly negatively correlated with MDS15 risk scores (*p* = 2.15e-3, Pearson’s coefficient = −0.28, Supplementary Figure S10), suggesting the association of MDS15 model with dysregulated immunome. The signaling analysis shed light on potential therapeutic targets for MDS transformation, paving the way for future drug development.

The primary innovation of the MDS15 lies in its enhanced prognostic accuracy compared to traditional systems like IPSS and WPSS. This enhanced precision can assist clinicians in identifying truly high-risk patients, enabling them to implement more proactive measures such as increased monitoring or interventions that can alter the disease’s natural progression, such as demethylating agents. Furthermore, by incorporating fewer variables in the MDS15, costs can be significantly reduced in contrast to comprehensive genome transcriptome analyses. Despite our study’s insights, there are limitations. The patient populations from GSE15061 and GSE58831 displayed heterogeneity in terms of age, diagnostic subtype, and various clinical and genetic variables (Mills et al., 2009; Gerstung et al., 2015). And the treatment profile was not described in both sets, which may produce potential bias. Moving forward, it will be imperative to develop a refined model that incorporates individualized patient information, and this should be validated using larger cohort studies.

## Conclusion

By the updated transcriptomic analysis method, WGCNA, leukemic transformation correlated gene cluster was identified, and a novel prediction model (MDS15) was established by LASSO. Then, the predictive power was shown to be superior to that of traditional prognostic systems (IPSS, WPSS, etc.), which was validated by datasets derived from different cell origins and independent cohorts. Moreover, disrupted MYC/E2F signaling, and inflammatory pathway were demonstrated to be associating with MDS15 risk scores.

## Data Availability

The original contributions presented in the study are included in the article/Supplementary Material, further inquiries can be directed to the corresponding author.

## Abbreviations

AML, acute myeloid leukemia; MDS, myelodysplastic syndrome; CMML, chronic myelomonocytic leukemia; RAEB, refractory anemia with excessive blasts; RARS, refractory anemia with ring sideroblasts; LFS, leukemia-free survival; WGCNA, weighted gene co-expression network analysis; LASSO, least absolute shrinkage and selection operator; IPSS, international prognostic scoring system; WPSS, WHO prognostic scoring system; ROC, receiver operating characteristic curve; AUC, area under curve; GSEA, gene set enrichment analysis; GEO, Gene Expression Omnibus; ORA, over-representation analysis.

## References

[B1] Adebayo O. O. Dammer E. B. Dill C. D. Adebayo A. O. Oseni S. O. Griffen T. L. (2022). Multivariant transcriptome analysis identifies modules and hub genes associated with poor outcomes in newly diagnosed multiple myeloma patients. Cancers (Basel) 14 (9), 2228. 10.3390/cancers14092228 35565356 PMC9104534

[B2] Aly M. Ramdzan Z. M. Nagata Y. Balasubramanian S. K. Hosono N. Makishima H. (2019). Distinct clinical and biological implications of CUX1 in myeloid neoplasms. Blood Adv. 3 (14), 2164–2178. 10.1182/bloodadvances.2018028423 31320321 PMC6650742

[B3] An N. Khan S. Imgruet M. K. Gurbuxani S. K. Konecki S. N. Burgess M. R. (2018). Gene dosage effect of CUX1 in a murine model disrupts HSC homeostasis and controls the severity and mortality of MDS. Blood 131 (24), 2682–2697. 10.1182/blood-2017-10-810028 29592892 PMC6032890

[B4] Avdic V. Zhang P. Lanouette S. Groulx A. Tremblay V. Brunzelle J. (2011). Structural and biochemical insights into MLL1 core complex assembly. Structure 19 (1), 101–108. 10.1016/j.str.2010.09.022 21220120

[B5] Bracken A. P. Pasini D. Capra M. Prosperini E. Colli E. Helin K. (2003). EZH2 is downstream of the pRB-E2F pathway, essential for proliferation and amplified in cancer. EMBO J. 22 (20), 5323–5335. 10.1093/emboj/cdg542 14532106 PMC213796

[B6] Chen W. Liang W. He Y. Liu C. Chen H. Lv P. (2022). Immune microenvironment-related gene mapping predicts immunochemotherapy response and prognosis in diffuse large B-cell lymphoma. Med. Oncol. 39 (4), 44. 10.1007/s12032-021-01642-3 35092504

[B7] Daver N. Schlenk R. F. Russell N. H. Levis M. J. (2019). Targeting FLT3 mutations in AML: review of current knowledge and evidence. Leukemia 33 (2), 299–312. 10.1038/s41375-018-0357-9 30651634 PMC6365380

[B8] Dimicoli S. Wei Y. Bueso-Ramos C. Yang H. Dinardo C. Jia Y. (2013). Overexpression of the toll-like receptor (TLR) signaling adaptor MYD88, but lack of genetic mutation, in myelodysplastic syndromes. PLoS One 8 (8), e71120. 10.1371/journal.pone.0071120 23976989 PMC3744562

[B9] Dou Y. Milne T. A. Tackett A. J. Smith E. R. Fukuda A. Wysocka J. (2005). Physical association and coordinate function of the H3 K4 methyltransferase MLL1 and the H4 K16 acetyltransferase MOF. Cell 121 (6), 873–885. 10.1016/j.cell.2005.04.031 15960975

[B10] Drabkin H. A. Parsy C. Ferguson K. Guilhot F. Lacotte L. Roy L. (2002). Quantitative HOX expression in chromosomally defined subsets of acute myelogenous leukemia. Leukemia 16 (2), 186–195. 10.1038/sj.leu.2402354 11840284

[B11] Dweep H. Sticht C. Gretz N. (2013). In-silico algorithms for the screening of possible microRNA binding sites and their interactions. Curr. Genomics 14 (2), 127–136. 10.2174/1389202911314020005 24082822 PMC3637677

[B12] Edgar R. Domrachev M. Lash A. E. (2002). Gene Expression Omnibus: NCBI gene expression and hybridization array data repository. Nucleic Acids Res. 30 (1), 207–210. 10.1093/nar/30.1.207 11752295 PMC99122

[B13] Feng S. Liu N. Chen X. Liu Y. An J. (2020). Long non-coding RNA NEAT1/miR-338-3p axis impedes the progression of acute myeloid leukemia via regulating CREBRF. Cancer Cell Int. 20, 112. 10.1186/s12935-020-01182-2 32280304 PMC7137299

[B14] Fu Y. Xu M. Cui Z. Yang Z. Zhang Z. Yin X. (2020). Genome-wide identification of FHL1 as a powerful prognostic candidate and potential therapeutic target in acute myeloid leukaemia. EBioMedicine 52, 102664. 10.1016/j.ebiom.2020.102664 32062360 PMC7021551

[B15] Gajzer D. Logothetis C. N. Sallman D. A. Calon G. Babu A. Chan O. (2021). MYC overexpression is associated with an early disease progression from MDS to AML. Leuk. Res. 111, 106733. 10.1016/j.leukres.2021.106733 34749168 PMC8643343

[B16] Gelsi-Boyer V. Trouplin V. Adelaide J. Bonansea J. Cervera N. Carbuccia N. (2009). Mutations of polycomb-associated gene ASXL1 in myelodysplastic syndromes and chronic myelomonocytic leukaemia. Br. J. Haematol. 145 (6), 788–800. 10.1111/j.1365-2141.2009.07697.x 19388938

[B17] Gerstung M. Pellagatti A. Malcovati L. Giagounidis A. Porta M. G. Jadersten M. (2015). Combining gene mutation with gene expression data improves outcome prediction in myelodysplastic syndromes. Nat. Commun. 6, 5901. 10.1038/ncomms6901 25574665 PMC4338540

[B18] Greenberg P. Cox C. LeBeau M. M. Fenaux P. Morel P. Sanz G. (1997). International scoring system for evaluating prognosis in myelodysplastic syndromes. Blood 89 (6), 2079–2088. 10.1182/blood.v89.6.2079 9058730

[B19] Greenberg P. L. Tuechler H. Schanz J. Sanz G. Garcia-Manero G. Sole F. (2012). Revised international prognostic scoring system for myelodysplastic syndromes. Blood 120 (12), 2454–2465. 10.1182/blood-2012-03-420489 22740453 PMC4425443

[B20] He Z. Zhang S. Ma D. Fang Q. Yang L. Shen S. (2019). HO-1 promotes resistance to an EZH2 inhibitor through the pRB-E2F pathway: correlation with the progression of myelodysplastic syndrome into acute myeloid leukemia. J. Transl. Med. 17 (1), 366. 10.1186/s12967-019-2115-9 31711520 PMC6849246

[B21] Heinrichs S. Conover L. F. Bueso-Ramos C. E. Kilpivaara O. Stevenson K. Neuberg D. (2013). MYBL2 is a sub-haploinsufficient tumor suppressor gene in myeloid malignancy. Elife 2, e00825. 10.7554/eLife.00825 23878725 PMC3713455

[B22] Hemmati S. Haque T. Gritsman K. (2017). Inflammatory signaling pathways in preleukemic and leukemic stem cells. Front. Oncol. 7, 265. 10.3389/fonc.2017.00265 29181334 PMC5693908

[B23] Huang da W. Sherman B. T. Lempicki R. A. (2009). Systematic and integrative analysis of large gene lists using DAVID bioinformatics resources. Nat. Protoc. 4 (1), 44–57. 10.1038/nprot.2008.211 19131956

[B24] Huang H. H. Chen F. Y. Chou W. C. Hou H. A. Ko B. S. Lin C. T. (2019). Long non-coding RNA HOXB-AS3 promotes myeloid cell proliferation and its higher expression is an adverse prognostic marker in patients with acute myeloid leukemia and myelodysplastic syndrome. BMC Cancer 19 (1), 617. 10.1186/s12885-019-5822-y 31234830 PMC6591843

[B25] Janusz K. Izquierdo M. M. Cadenas F. L. Ramos F. Sanchez J. M. H. Lumbreras E. (2021). Clinical, biological, and prognostic implications of SF3B1 co-occurrence mutations in very low/low- and intermediate-risk MDS patients. Ann. Hematol. 100 (8), 1995–2004. 10.1007/s00277-020-04360-4 33409621

[B26] Jin P. Tan Y. Zhang W. Li J. Wang K. (2020). Prognostic alternative mRNA splicing signatures and associated splicing factors in acute myeloid leukemia. Neoplasia 22 (9), 447–457. 10.1016/j.neo.2020.06.004 32653835 PMC7356271

[B27] Kerbauy D. B. Deeg H. J. (2007). Apoptosis and antiapoptotic mechanisms in the progression of myelodysplastic syndrome. Exp. Hematol. 35 (11), 1739–1746. 10.1016/j.exphem.2007.09.007 17976524 PMC2131709

[B28] Kondo Y. Hirakawa Y. Kieber J. J. Fukuda H. (2011). CLE peptides can negatively regulate protoxylem vessel formation via cytokinin signaling. Plant Cell Physiol. 52 (1), 37–48. 10.1093/pcp/pcq129 20802224 PMC3023848

[B29] Kotani S. Yoda A. Kon A. Kataoka K. Ochi Y. Shiozawa Y. (2019). Molecular pathogenesis of disease progression in MLL-rearranged AML. Leukemia 33 (3), 612–624. 10.1038/s41375-018-0253-3 30209403 PMC6462875

[B30] Langfelder P. Horvath S. (2008). WGCNA: an R package for weighted correlation network analysis. BMC Bioinforma. 9, 559. 10.1186/1471-2105-9-559 PMC2631488 19114008

[B31] Li A. Yang Y. Gao C. Lu J. Jeong H. W. Liu B. H. (2013). A SALL4/MLL/HOXA9 pathway in murine and human myeloid leukemogenesis. J. Clin. Invest. 123 (10), 4195–4207. 10.1172/JCI62891 24051379 PMC3784519

[B32] Li J. He F. Zhang P. Chen S. Shi H. Sun Y. (2017). Loss of Asxl2 leads to myeloid malignancies in mice. Nat. Commun. 8, 15456. 10.1038/ncomms15456 28593990 PMC5472177

[B33] Lin W. Y. Fordham S. E. Hungate E. Sunter N. J. Elstob C. Xu Y. (2021). Genome-wide association study identifies susceptibility loci for acute myeloid leukemia. Nat. Commun. 12 (1), 6233. 10.1038/s41467-021-26551-x 34716350 PMC8556284

[B34] Lindsley R. C. Ebert B. L. (2013). Molecular pathophysiology of myelodysplastic syndromes. Annu. Rev. Pathol. 8, 21–47. 10.1146/annurev-pathol-011811-132436 22934674 PMC3514602

[B35] Liquori A. Ibanez M. Sargas C. Sanz M. A. Barragan E. Cervera J. (2020). Acute promyelocytic leukemia: a constellation of molecular events around a single PML-RARA fusion gene. Cancers (Basel) 12 (3), 624. 10.3390/cancers12030624 32182684 PMC7139833

[B36] Liu H. Liu M. You H. Li X. Li X. (2020). Oncogenic network and hub genes for natural killer/T-cell lymphoma utilizing WGCNA. Front. Oncol. 10, 223. 10.3389/fonc.2020.00223 32195177 PMC7066115

[B37] Malcovati L. Germing U. Kuendgen A. Della Porta M. G. Pascutto C. Invernizzi R. (2007). Time-dependent prognostic scoring system for predicting survival and leukemic evolution in myelodysplastic syndromes. J. Clin. Oncol. 25 (23), 3503–3510. 10.1200/JCO.2006.08.5696 17687155

[B38] Mayer I. A. Verma A. Grumbach I. M. Uddin S. Lekmine F. Ravandi F. (2001). The p38 MAPK pathway mediates the growth inhibitory effects of interferon-alpha in BCR-ABL-expressing cells. J. Biol. Chem. 276 (30), 28570–28577. 10.1074/jbc.M011685200 11353767

[B39] Mills K. I. Kohlmann A. Williams P. M. Wieczorek L. Liu W. M. Li R. (2009). Microarray-based classifiers and prognosis models identify subgroups with distinct clinical outcomes and high risk of AML transformation of myelodysplastic syndrome. Blood 114 (5), 1063–1072. 10.1182/blood-2008-10-187203 19443663

[B40] Mochizuki-Kashio M. Aoyama K. Sashida G. Oshima M. Tomioka T. Muto T. (2015). Ezh2 loss in hematopoietic stem cells predisposes mice to develop heterogeneous malignancies in an Ezh1-dependent manner. Blood 126 (10), 1172–1183. 10.1182/blood-2015-03-634428 26219303

[B41] Mootha V. K. Lindgren C. M. Eriksson K. F. Subramanian A. Sihag S. Lehar J. (2003). PGC-1alpha-responsive genes involved in oxidative phosphorylation are coordinately downregulated in human diabetes. Nat. Genet. 34 (3), 267–273. 10.1038/ng1180 12808457

[B42] Muto T. Guillamot M. Yeung J. Fang J. Bennett J. Nadorp B. (2022). TRAF6 functions as a tumor suppressor in myeloid malignancies by directly targeting MYC oncogenic activity. Cell Stem Cell 29 (2), 298–314.e9. 10.1016/j.stem.2021.12.007 35045331 PMC8822959

[B43] Nagata Y. Maciejewski J. P. (2019). The functional mechanisms of mutations in myelodysplastic syndrome. Leukemia 33 (12), 2779–2794. 10.1038/s41375-019-0617-3 31673113 PMC8370479

[B44] Nakamura T. Mori T. Tada S. Krajewski W. Rozovskaia T. Wassell R. (2002). ALL-1 is a histone methyltransferase that assembles a supercomplex of proteins involved in transcriptional regulation. Mol. Cell 10 (5), 1119–1128. 10.1016/s1097-2765(02)00740-2 12453419

[B45] Nguyen H. D. Leong W. Y. Li W. Reddy P. N. G. Sullivan J. D. Walter M. J. (2018). Spliceosome mutations induce R loop-associated sensitivity to ATR inhibition in myelodysplastic syndromes. Cancer Res. 78 (18), 5363–5374. 10.1158/0008-5472.CAN-17-3970 30054334 PMC6139047

[B46] Paracatu L. C. Schuettpelz L. G. (2020). Contribution of aberrant toll like receptor signaling to the pathogenesis of myelodysplastic syndromes. Front. Immunol. 11, 1236. 10.3389/fimmu.2020.01236 32625214 PMC7313547

[B47] Patel A. Dharmarajan V. Vought V. E. Cosgrove M. S. (2009). On the mechanism of multiple lysine methylation by the human mixed lineage leukemia protein-1 (MLL1) core complex. J. Biol. Chem. 284 (36), 24242–24256. 10.1074/jbc.M109.014498 19556245 PMC2782018

[B48] Pellagatti A. Cazzola M. Giagounidis A. Perry J. Malcovati L. Della Porta M. G. (2010). Deregulated gene expression pathways in myelodysplastic syndrome hematopoietic stem cells. Leukemia 24 (4), 756–764. 10.1038/leu.2010.31 20220779

[B49] Pellagatti A. Cazzola M. Giagounidis A. A. Malcovati L. Porta M. G. Killick S. (2006). Gene expression profiles of CD34+ cells in myelodysplastic syndromes: involvement of interferon-stimulated genes and correlation to FAB subtype and karyotype. Blood 108 (1), 337–345. 10.1182/blood-2005-12-4769 16527891

[B50] Platzbecker U. Kubasch A. S. Homer-Bouthiette C. Prebet T. (2021). Current challenges and unmet medical needs in myelodysplastic syndromes. Leukemia 35 (8), 2182–2198. 10.1038/s41375-021-01265-7 34045662 PMC8324480

[B51] Rautenberg C. Germing U. Pechtel S. Lamers M. Fischermanns C. Jager P. (2019). Prognostic impact of peripheral blood WT1-mRNA expression in patients with MDS. Blood Cancer J. 9 (11), 86. 10.1038/s41408-019-0248-y 31719523 PMC6851368

[B52] Rostami M. Kharajo R. S. Parsa-Kondelaji M. Ayatollahi H. Sheikhi M. Keramati M. R. (2022). Altered expression of NEAT1 variants and P53, PTEN, and BCL-2 genes in patients with acute myeloid leukemia. Leuk. Res. 115, 106807. 10.1016/j.leukres.2022.106807 35231756

[B53] Sakhdari A. Class C. Montalban-Bravo G. Sasaki K. Bueso-Ramos C. E. Patel K. P. (2022). Immunohistochemical loss of enhancer of Zeste Homolog 2 (EZH2) protein expression correlates with EZH2 alterations and portends a worse outcome in myelodysplastic syndromes. Mod. Pathol. 35, 1212–1219. 10.1038/s41379-022-01074-y 35504958 PMC13105204

[B54] Snoeck H. W. Van Bockstaele D. R. Nys G. Lenjou M. Lardon F. Haenen L. (1994). Interferon gamma selectively inhibits very primitive CD342+CD38-and not more mature CD34+CD38+ human hematopoietic progenitor cells. J. Exp. Med. 180 (3), 1177–1182. 10.1084/jem.180.3.1177 7520470 PMC2191667

[B55] Subramanian A. Tamayo P. Mootha V. K. Mukherjee S. Ebert B. L. Gillette M. A. (2005). Gene set enrichment analysis: a knowledge-based approach for interpreting genome-wide expression profiles. Proc. Natl. Acad. Sci. U. S. A. 102 (43), 15545–15550. 10.1073/pnas.0506580102 16199517 PMC1239896

[B56] Tsai H. K. Gibson C. J. Murdock H. M. Davineni P. K. Harris M. Wang E. S. (2022). Allelic complexity of KMT2A partial tandem duplications in acute myeloid leukemia and myelodysplastic syndromes. Blood Adv. 6, 4236–4240. 10.1182/bloodadvances.2022007613 35584376 PMC9327559

[B57] Vasquez M. M. Hu C. Roe D. J. Chen Z. Halonen M. Guerra S. (2016). Least absolute shrinkage and selection operator type methods for the identification of serum biomarkers of overweight and obesity: simulation and application. BMC Med. Res. Methodol. 16 (1), 154. 10.1186/s12874-016-0254-8 27842498 PMC5109787

[B58] Walter M. J. Ding L. Shen D. Shao J. Grillot M. McLellan M. (2011). Recurrent DNMT3A mutations in patients with myelodysplastic syndromes. Leukemia 25 (7), 1153–1158. 10.1038/leu.2011.44 21415852 PMC3202965

[B59] Wei Y. Dimicoli S. Bueso-Ramos C. Chen R. Yang H. Neuberg D. (2013). Toll-like receptor alterations in myelodysplastic syndrome. Leukemia 27 (9), 1832–1840. 10.1038/leu.2013.180 23765228 PMC4011663

[B60] Xu F. Zhu Y. He Q. Wu L. Y. Zhang Z. Shi W. H. (2016). Identification of microRNA-regulated pathways using an integration of microRNA-mRNA microarray and bioinformatics analysis in CD34+ cells of myelodysplastic syndromes. Sci. Rep. 6, 32232. 10.1038/srep32232 27571714 PMC5004188

[B61] Yan H. Wang Z. Sun Y. Hu L. Bu P. (2021). Cytoplasmic NEAT1 suppresses AML stem cell self-renewal and leukemogenesis through inactivation of wnt signaling. Adv. Sci. (Weinh) 8 (22), e2100914. 10.1002/advs.202100914 34609794 PMC8596104

[B62] Zhang B. Horvath S. (2005). A general framework for weighted gene co-expression network analysis. Stat. Appl. Genet. Mol. Biol. 4, Article17. 10.2202/1544-6115.1128 16646834

[B63] Zhao C. Wang S. Zhao Y. Du F. Wang W. Lv P. (2019). Long noncoding RNA NEAT1 modulates cell proliferation and apoptosis by regulating miR-23a-3p/SMC1A in acute myeloid leukemia. J. Cell Physiol. 234 (5), 6161–6172. 10.1002/jcp.27393 30246348

[B64] Zhong W. J. Liu X. D. Zhong L. Y. Li K. B. Sun Q. X. Xu X. (2021). Comparison of gene mutation spectra in younger and older Chinese acute myeloid leukemia patients and its prognostic value. Gene 770, 145344. 10.1016/j.gene.2020.145344 33333221

[B65] Zhou H. Liu W. Zhou Y. Hong Z. Ni J. Zhang X. (2021). Therapeutic inhibition of GAS6-AS1/YBX1/MYC axis suppresses cell propagation and disease progression of acute myeloid leukemia. J. Exp. Clin. Cancer Res. 40 (1), 353. 10.1186/s13046-021-02145-9 34753494 PMC8576903

