# Supplementary figures and images for: Predicting leukemic transformation in myelodysplastic syndrome using a transcriptomic signature
Source: Front Genet. 2023 Oct 25;14:1235315. doi: 10.3389/fgene.2023.1235315 (PMC10634373; doi:10.3389/fgene.2023.1235315)

A

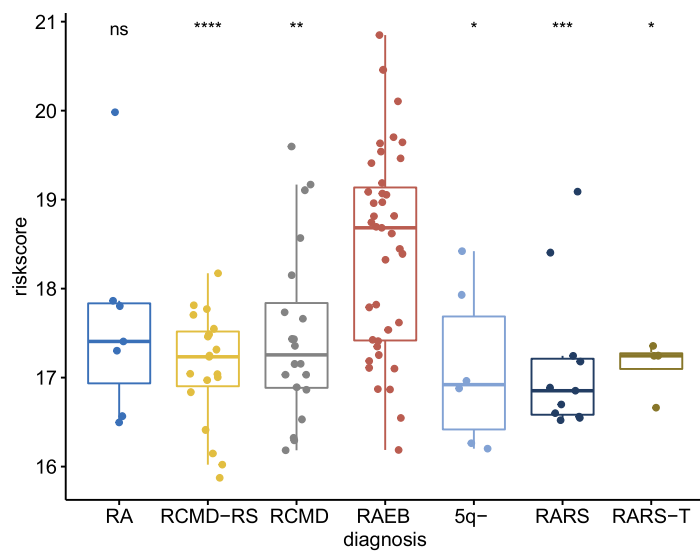

B

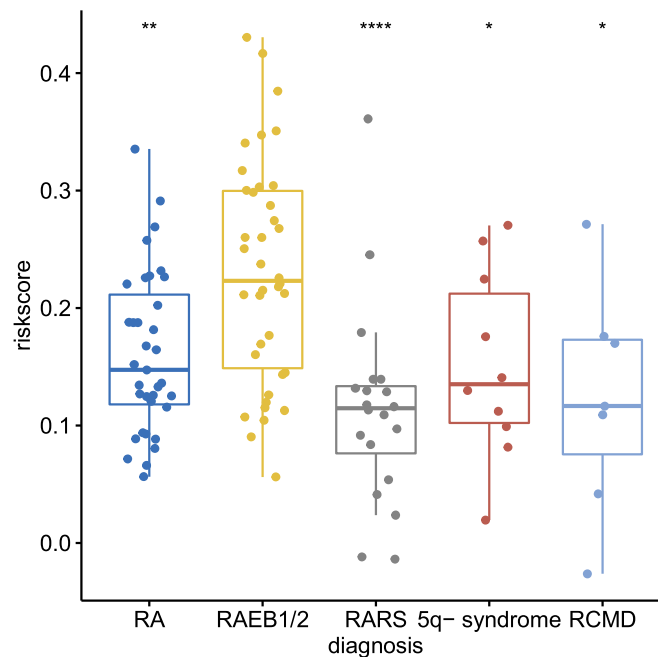

C

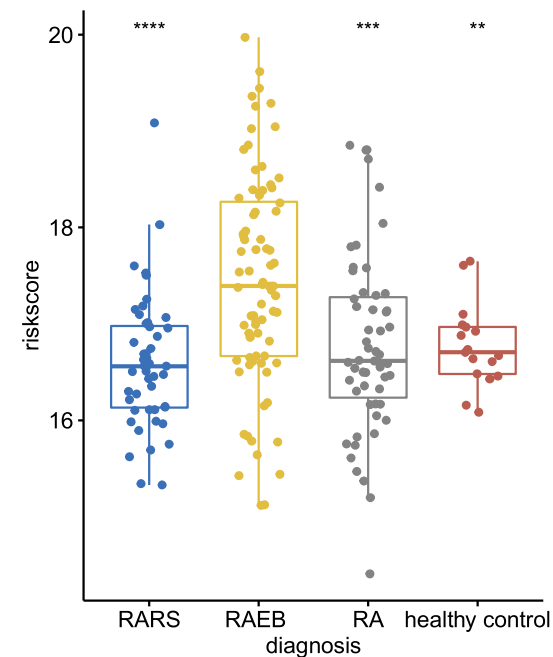

D

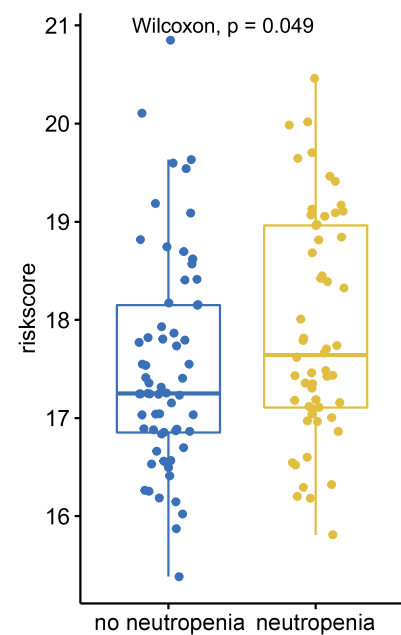

E

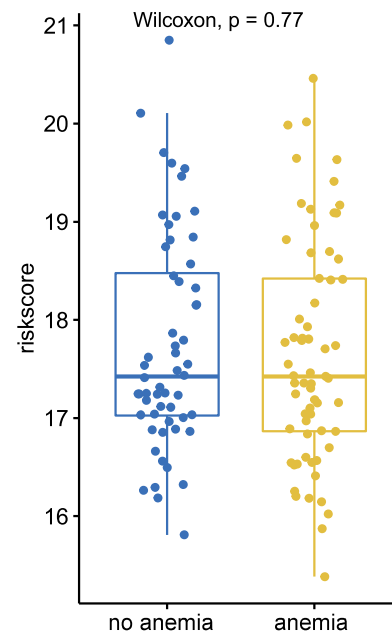

F

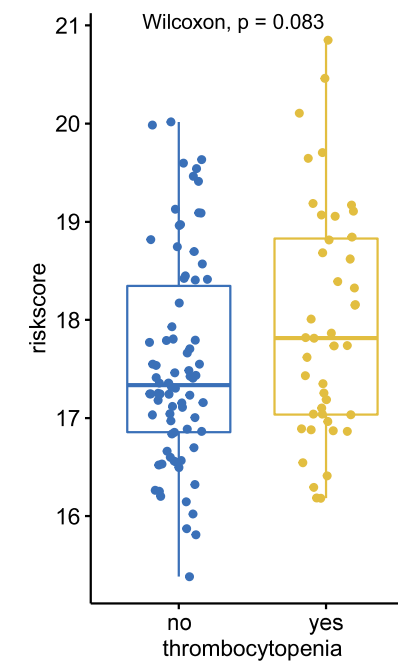

G

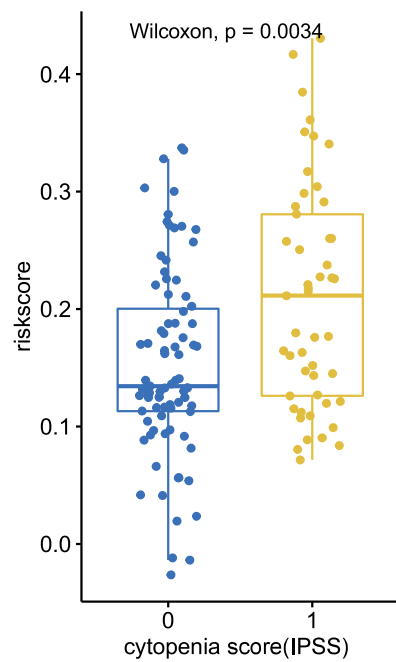

H

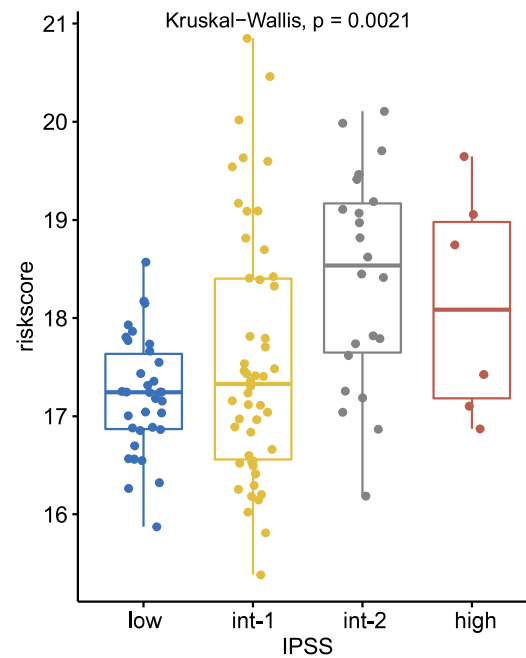

I

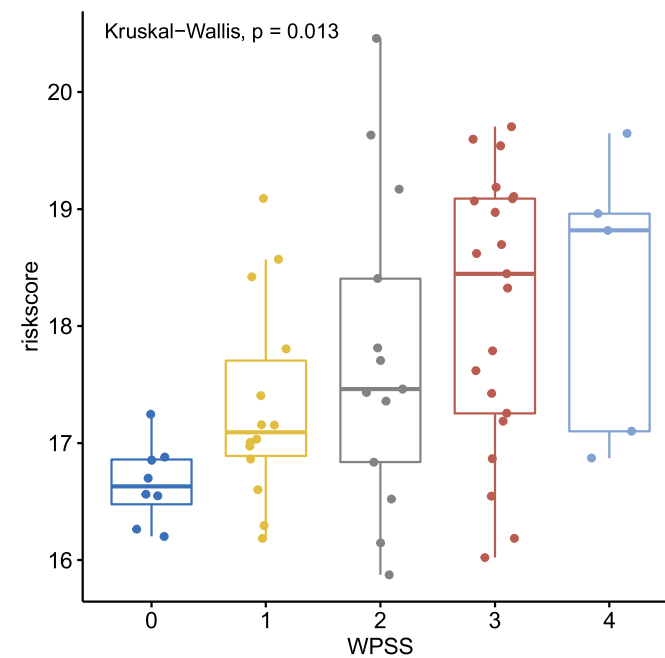

J

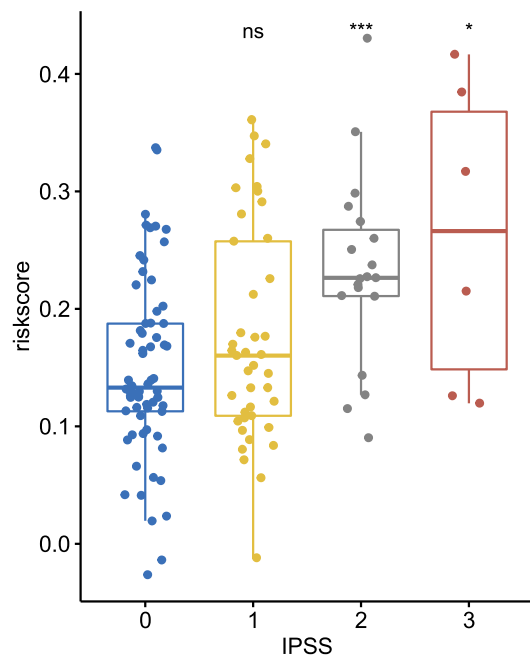

K

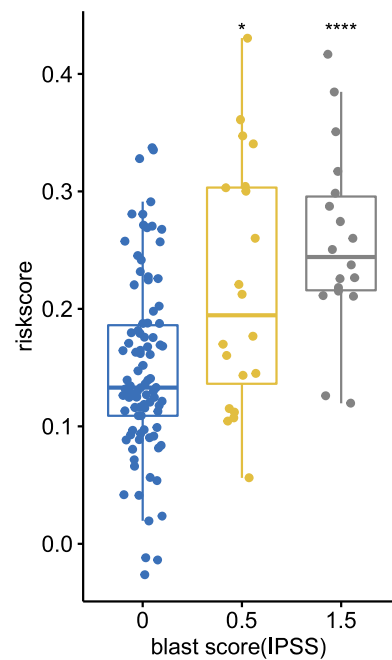

Supplement: Supplementary file 1 [file DataSheet7.PDF]

Sample dendrogram and trait heatmap

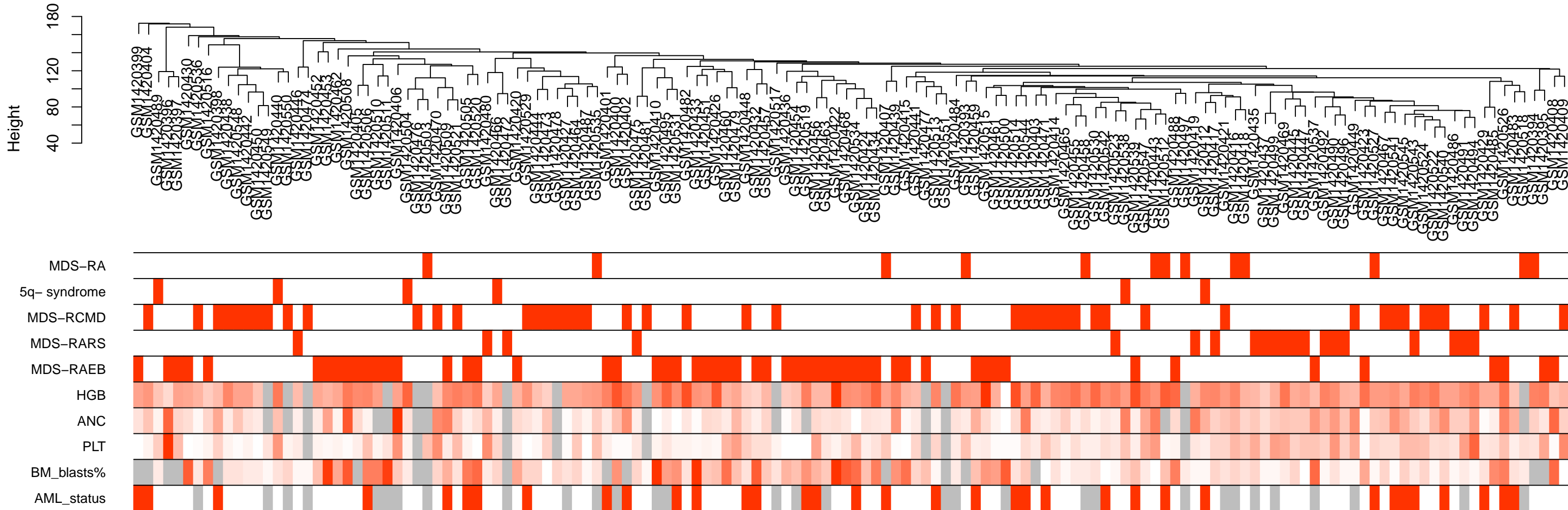

Supplement: Supplementary file 2 [file DataSheet2.PDF]

# Cluster Dendrogram

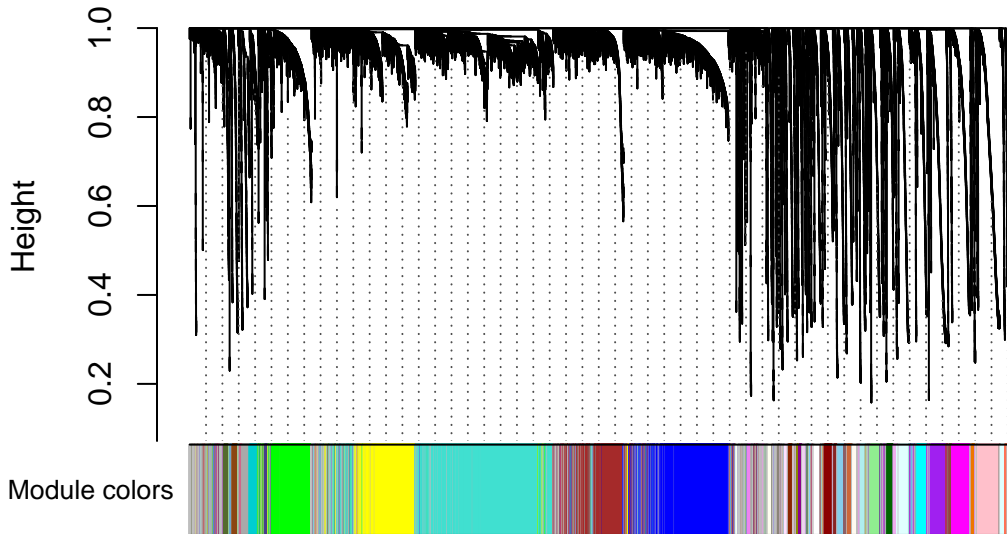

Supplement: Supplementary file 3 [file DataSheet4.PDF]

A

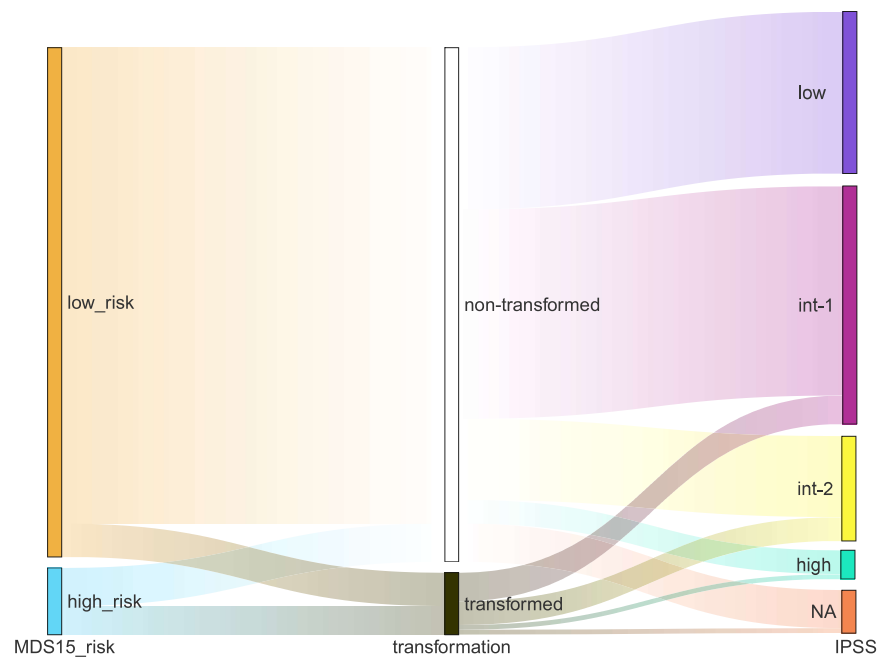

B

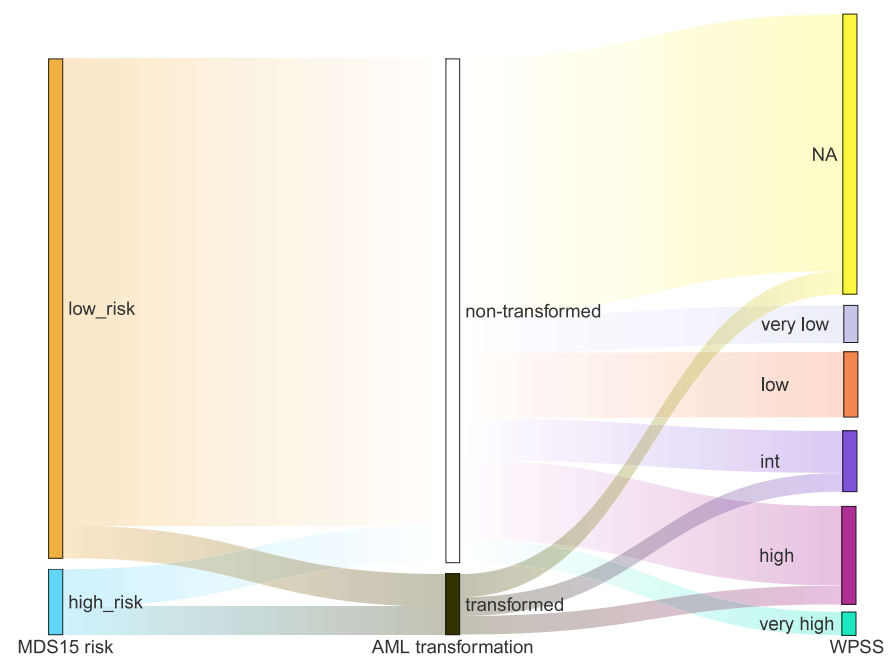

Supplement: Supplementary file 4 [file DataSheet6.PDF]

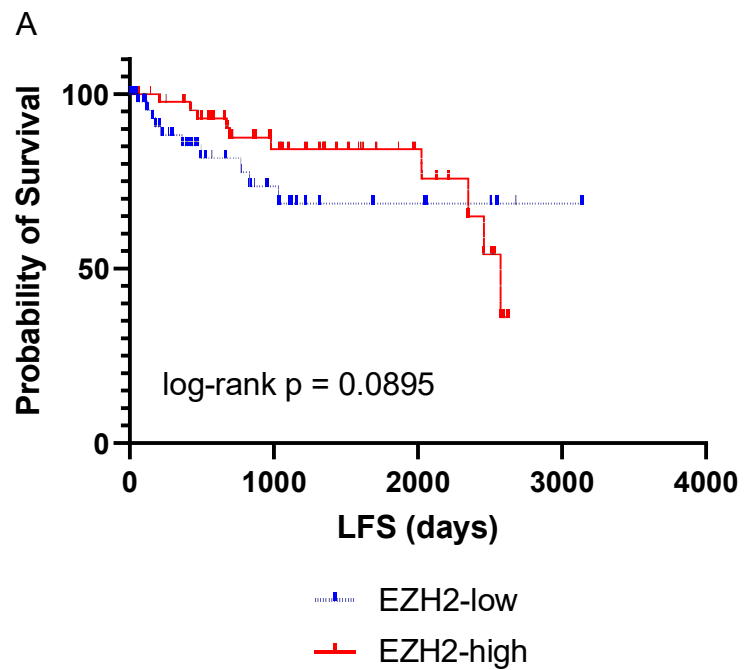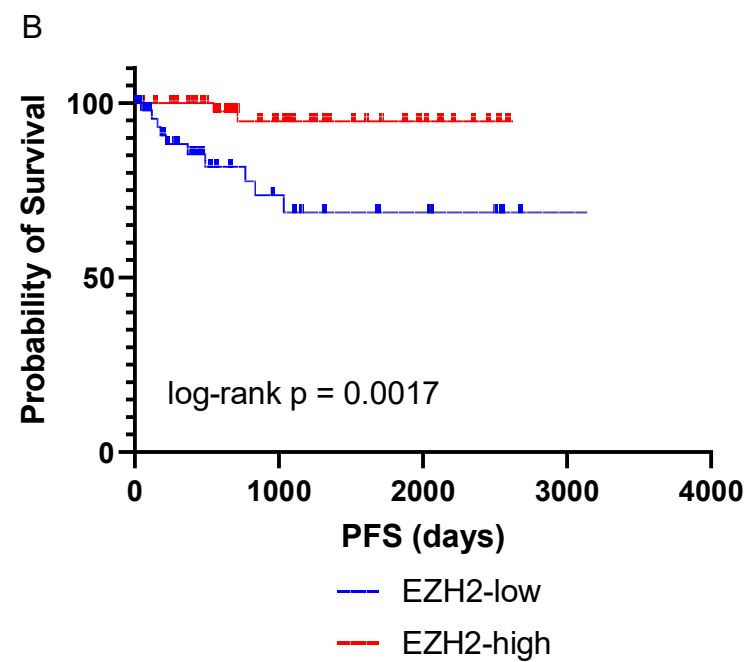

Supplement: Supplementary file 5 [file DataSheet9.PDF]

### Scale independence

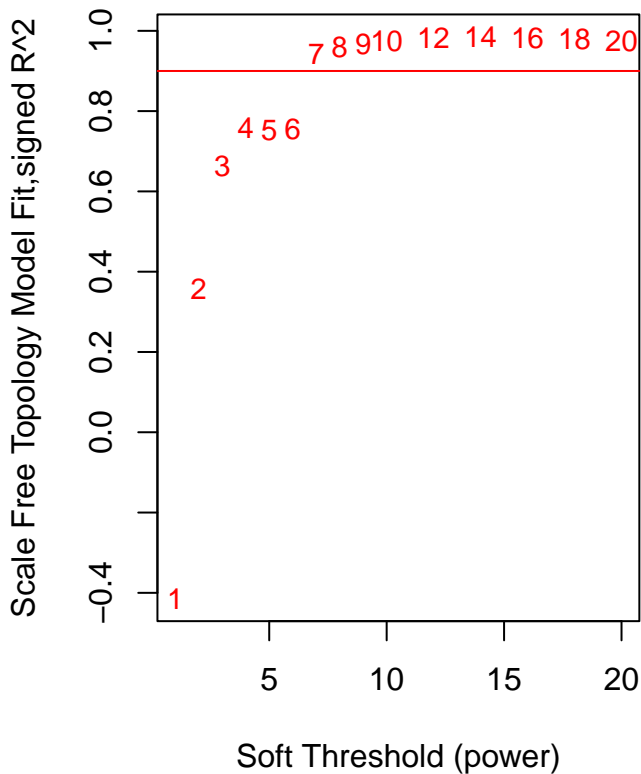

### Mean connectivity

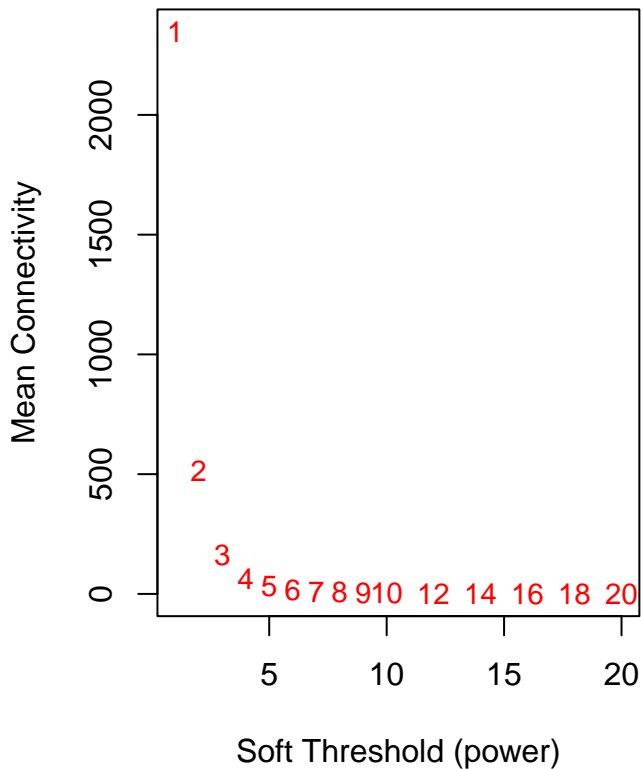

Supplement: Supplementary file 6 [file DataSheet3.PDF]

Sample clustering to detect outliers

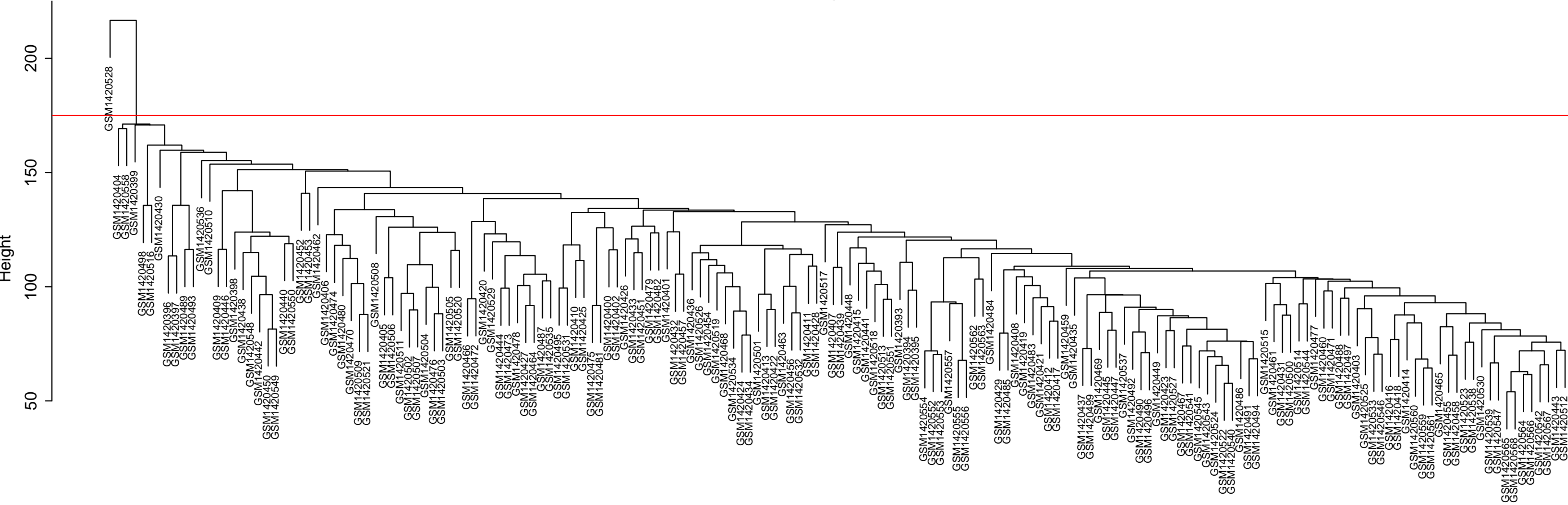

Supplement: Supplementary file 7 [file DataSheet1.PDF]

**Module membership vs. gene significance**  
**cor=0.31, p=2.2e-30**

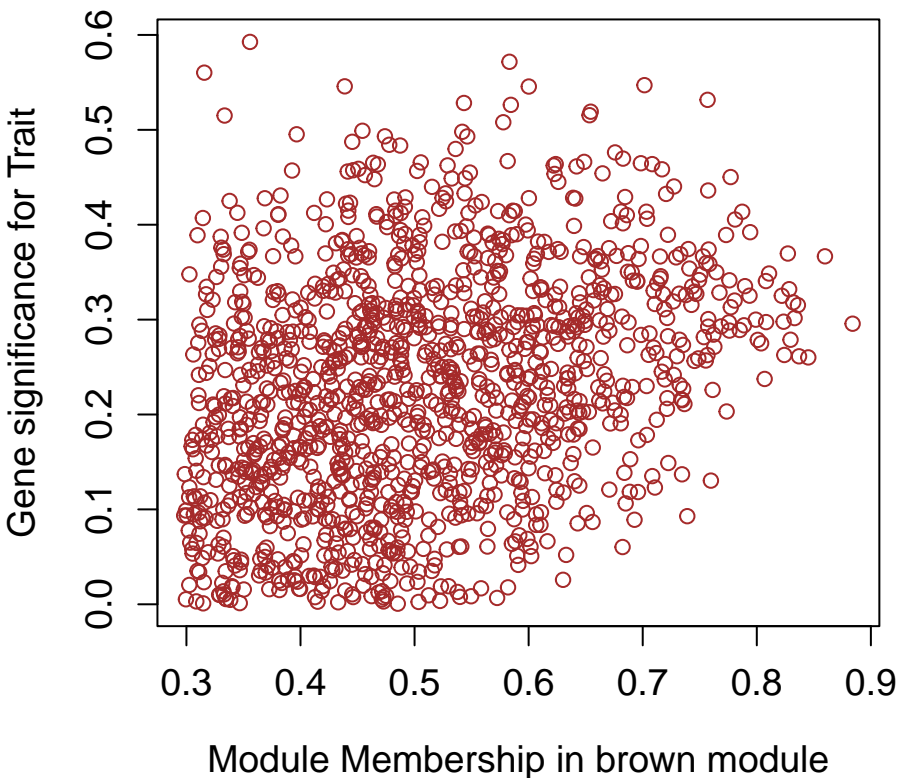

Supplement: Supplementary file 8 [file DataSheet5.PDF]
