# Supplementary material for: Predicting leukemic transformation in myelodysplastic syndrome using a transcriptomic signature
Source: Front Genet. 2023 Oct 25;14:1235315. doi: 10.3389/fgene.2023.1235315 (PMC10634373; doi:10.3389/fgene.2023.1235315)
Supplement: Supplementary file 10 [file DataSheet8.PDF]

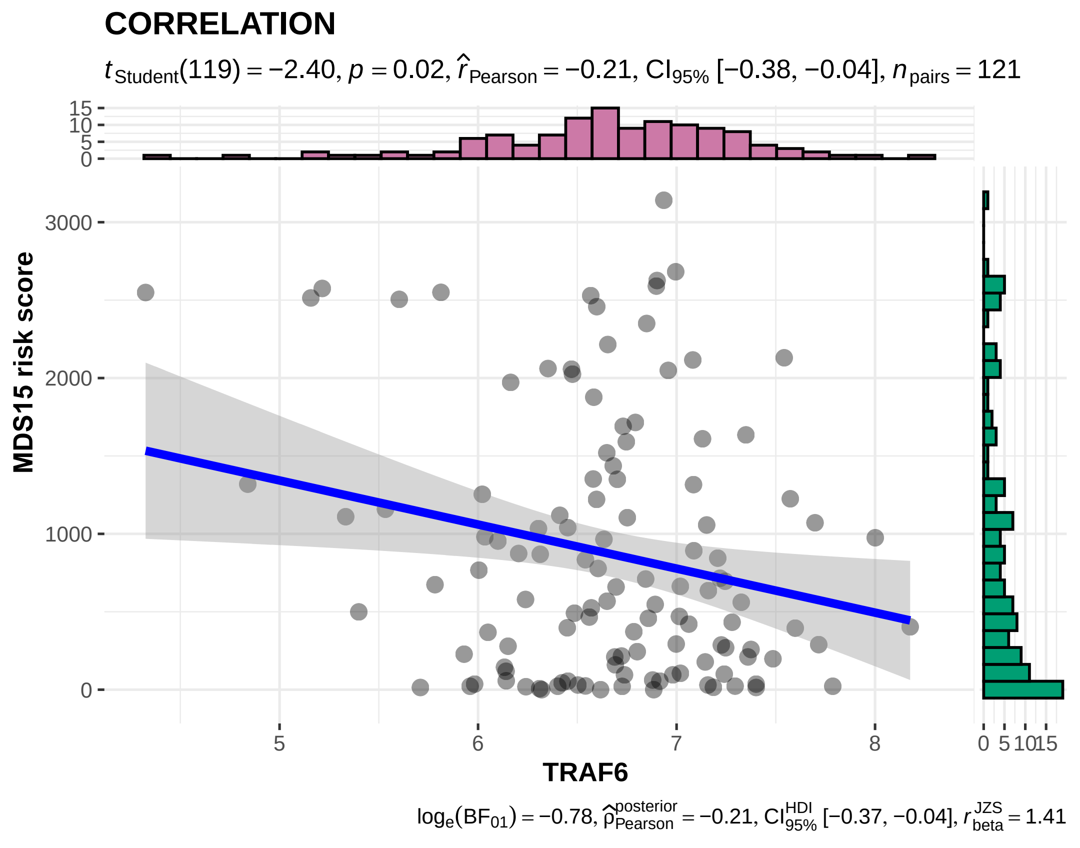

CORRELATION
$t_{\text{Student}}(119) = -2.40$, $p = 0.02$, $\hat{r}_{\text{Pearson}} = -0.21$, $\text{CI}_{95\%}$ [−0.38, −0.04], $n_{\text{pairs}} = 121$
15
10
5
0
3000
2000
1000
0
MDS15 risk score
5
6
7
8
0
5
10
15
TRAF6
$\log_e(\text{BF}_{01}) = -0.78$, $\hat{\rho}^{\text{posterior}}_{\text{Pearson}} = -0.21$, $\text{CI}^{\text{HDI}}_{95\%}$ [−0.37, −0.04], $r^{\text{JZS}}_{\text{beta}} = 1.41$
