# Supplementary material for: Predicting leukemic transformation in myelodysplastic syndrome using a transcriptomic signature
Source: Front Genet. 2023 Oct 25;14:1235315. doi: 10.3389/fgene.2023.1235315 (PMC10634373; doi:10.3389/fgene.2023.1235315)
Supplement: Supplementary file 11 [file DataSheet10.PDF]

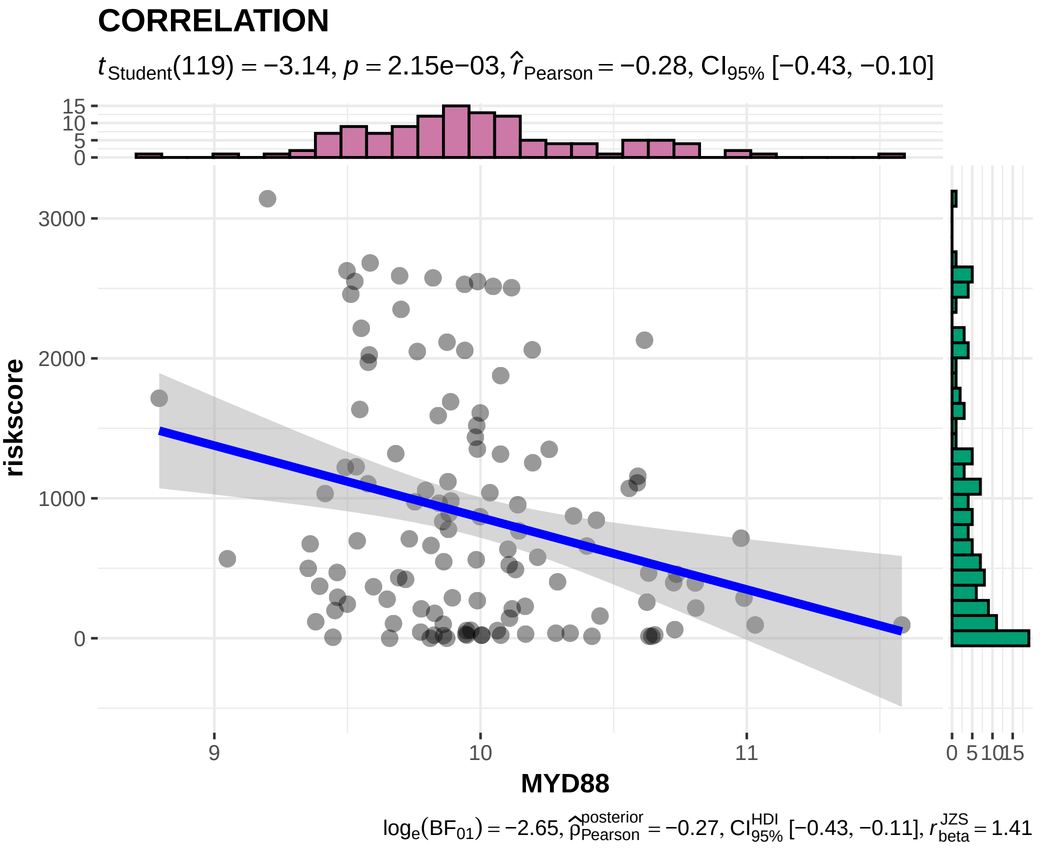
CORRELATION
$t_{Student}(119) = -3.14$, $p = 2.15e-03$, $\hat{r}_{Pearson} = -0.28$, $CI_{95\%}$ [−0.43, −0.10]
15
10
5
0
3000
2000
1000
0
riskscore
9
10
11
0 5 10 15
MYD88
$\log_e(BF_{01}) = -2.65$, $\hat{\rho}_{Pearson}^{posterior} = -0.27$, $CI_{95\%}^{HDI}$ [−0.43, −0.11], $r_{beta}^{JZS} = 1.41$
